# Differential morphology and distribution of GFAP astrocytes in vocal brain circuit in a songbird Southern house wren and humans

**DOI:** 10.3389/fnana.2026.1606172

**Published:** 2026-02-06

**Authors:** Santiago Hinestroza-Morales, Carolina López-Murillo, Hernán Hoyos-Maya, Geysson J. Fernandez, Andrés Villegas-Lanau, Hector Fabio Rivera-Gutierrez, Rafael Posada-Duque

**Affiliations:** 1 Área de Neurofisiología Celular, Grupo de Neurociencias de Antioquia, Facultad de Ciencias Exactas y Naturales, Instituto de Biología, Universidad de Antioquia, Medellín, Colombia; 2 Neurobanco, Grupo de Neurociencia de Antioquia, Facultad de Medicina, Universidad de Antioquia, Medellín, Colombia; 3 Grupo de Investigación de Ecología y Evolución de Vertebrados, Instituto de Biología, Universidad de Antioquia, Medellín, Colombia

**Keywords:** astrocytes, basal ganglia, GFAP, GS, human, song, Southern house wren, speech

## Abstract

Speech and song exhibit notable parallels between humans and birds. In humans, speech involves the Laryngeal Motor Cortex (LMC), Sensorimotor cortex (SMC), Broca’s and Wernicke’s areas, and the basal ganglia (striatum), which show convergent gene expression with avian song-control regions (RA, LMAN, HVC) and basal ganglia (Area X and medial striatum). While astrocyte morphology has been implicated in human speech, its role in song remains unknown. To compare astrocytes involved in speech and song, we evaluated cell density, astrocyte types, and their distribution in healthy humans and Southern house wrens using Nissl staining, GFAP and GS immunostaining, and 3D confocal imaging. The basal ganglia, human striatum and avian medial striatum, showed the highest cell density in both species. Human astrocyte distribution followed established cortical patterns, with enrichment in layers I–III and white matter (WM). In contrast, Southern house wrens exhibited restricted GFAP-positive astrocytes in vocal nuclei, with expression instead concentrated in telencephalic borders, vascular regions, and basal ganglia WM. Astrocyte morphology varied regionally in both species; basal ganglia astrocytes were especially complex, yet Southern house wrens exhibited reduced branching even after normalizing for brain volume/body weight ratio, indicating species-specific differences in complexity. GS-positive astrocytes were abundant and homogeneous throughout the pallium, including all vocal nuclei, unlike the more restricted GFAP-positive subset. Cross-species analysis of public songbird datasets confirmed minimal *GFAP* and strong *GLUL* (GS gene) expression in telencephalic astrocytes, opposite to humans, who show robust expression of both markers. Overall, GS astrocytes displayed a broadly uniform organization in both species, whereas GFAP astrocytes exhibited more restricted and enriched distributions, particularly in human speech-related basal ganglia, revealing species-specific differences in astrocyte architecture within vocal circuits.

## Highlights

- The region with the highest cell density in both humans and birds was the basal ganglia.
- GFAP-positive astrocytes in the vocal brain circuits of humans and Southern house wrens exhibited branched morphologies.
- In both species, GFAP astrocytes in the basal ganglia showed particularly extensive branching.
- Southern house wren GFAP astrocytes exhibited less branching than those observed in humans.
- Southern house wren GS-positive astrocytes constituted a relatively homogeneous population across both the pallium, exhibiting a comparable number of primary processes to those observed in human astrocytes.
- Cross-species analysis of public songbird datasets validated minimal *GFAP* and strong *GLUL* (GS) expression in telencephalic astrocytes, opposite to humans, who show robust expression of both markers.

## Introduction

Memory and vocal learning are essential behaviors in both birdsong and human speech (Beecher and Brenowitz, 2005). This rare trait, defined as the ability to produce learned, volitional vocalizations, is shared by a limited number of species, including humans, other vocal-learning mammals and certain birds such as hummingbirds, parrots, and songbirds (Bolhuis and Gahr, 2006; Nieder and Mooney, 2020). Among these, the Southern house wren (*Troglodytes musculus*), a passerine songbird, is notable for its complex vocalizations and its adaptability to environments close to human activity (Johnson, 1998; Mooney, 2009).

Songbirds and humans share convergent neural circuits for vocal communication, with song learning in birds requiring auditory input from a tutor (Gobes et al., 2010). Comparative work suggests that these parallels stem from deep structural homologies-duplicated pallial-basal ganglia-thalamic pathways-independently adapted for learned vocal behavior in both groups. At the molecular level, transcriptomic studies reveal strong convergence between human speech regions and songbird vocal nuclei, including shared regulatory programs and circuit-specific gene signatures (Chakraborty and Jarvis, 2015; Pfenning et al., 2014), supporting the idea that vocal learning relies on conserved neural architectures that became independently specialized in birds and mammals.

Astrocytes show substantial morphological diversity tightly linked to their function, with protoplasmic and fibrous forms representing two major canonical types (Bayraktar et al., 2020; Oberheim et al., 2012). In humans, protoplasmic astrocytes form highly branched, non-overlapping domains across cortical layers II–VI, enabling broad structural and metabolic interactions (Bayraktar et al., 2020; Köhler et al., 2021; Marín-Padilla, 1995; Matyash and Kettenmann, 2010; Morel et al., 2017; Morel et al., 2019). Their morphology–function coupling (Hol and Pekny, 2015) and underlies adaptive plasticity (Chaboub and Deneen, 2013; Dossi et al., 2018; Reisin and Colombo, 2002; Sosunov et al., 2014; Tabata, 2015; Verkhratsky et al., 2019; Zhang et al., 2016). Human astrocytes are also larger, more complex, and more responsive than rodent astrocytes (Falcone, 2022; Oberheim et al., 2009; Reisin and Colombo, 2002; Sosunov et al., 2014; Zhang et al., 2016), underscoring the importance of examining astrocyte structure across species. This framework provides essential context for understanding how astrocytes may contribute to the neural substrates of vocal behavior in birds and mammals. Common astrocyte markers include glial fibrillary acidic protein (GFAP), vimentin, and glutamine synthetase (GS) (Escartin et al., 2021; Steinman et al., 2013), with protoplasmic astrocytes primarily found in gray matter and fibrous types in WM (Hanani and Verkhratsky, 2021). Morphological changes in astrocytes can occur in response to environmental cues, injury, or neuroanatomical location (Guillamón-Vivancos et al., 2015). Yet, astrocyte diversity and morphology in birds-particularly those involved in complex behaviors like singing-remain underexplored.

GFAP identifies fibrous and structurally specialized astrocytes, typically associated with WM, perivascular regions, and cytoskeletal scaffolding; whereas GS, through its near-exclusive astrocytic expression of glutamine synthetase (Albrecht et al., 2010; Rose et al., 2013; Son et al., 2019), labels the broader metabolic population involved in glutamate–glutamine cycling, neurotransmitter clearance, and excitatory-inhibitory balance (Escartin et al., 2021; Hakvoort et al., 2017; Osipova et al., 2018; Petroff, 2002; Steinman et al., 2013; Tapiero et al., 2002). Because the evolutionary specialization of these subtypes remains unresolved and each marker reflects only part of astroglial diversity, integrating both GFAP and GS is essential for comparing astrocyte architecture between species, allowing detection of conserved features and species-specific specializations.

In birds, the song-control system includes two key pathways: the Song Motor Pathway (SMP) for vocal output, and the Anterior Forebrain Pathway (AFP) for learning and plasticity (Bolhuis and Gahr, 2006; Sizemore and Perkel, 2011). A central nucleus, HVC, connects these two circuits and is essential for generating learned vocalizations (Marler and Slabbekoorn, 2004). The HVC receives afferents mainly from auditory regions and projects to the Robust nucleus of the Arcopallium (RA) of the SMP and Area X of the AFP. Area X with LMAN processes the sound learning instructed by the tutor and a posterior part of the brain, where the HVC communicates with RA, the nucleus in charge of motor coordination, to produce learned vocalizations (Bolhuis and Gahr, 2006; Kato and Okanoya, 2010).

Similarly, in humans, the Laryngeal Motor and Sensorimotor Cortices (LMC and SMC), Broca’s and Wernicke’s areas, and the striatum (putamen and caudate) coordinate vocal learning and production (Jarvis, 2019; Kumar et al., 2016). Transcriptomic comparisons have shown strong gene expression similarities between the RA in birds and the LMC in humans, and between Area X in birds and the striatum in humans (Gedman et al., 2022). Though Broca’s and Wernicke’s areas lack direct molecular homology with LMAN and HVC, they may share functional analogies (Gedman et al., 2022; Nieder and Mooney, 2020; Pfenning et al., 2014).

Despite well-documented neuronal organization in the songbird brain (Moorman et al., 2011), few studies have examined astrocyte morphology and distribution in vocal regions, and existing research primarily focuses on migratory or non-passerine species (Carvalho-Paulo et al., 2018). Recent single-cell sequencing has found parallels between astrocytes in the bird RA and those in human cortical layers V–VI, as well as between HVC and all human cortical layers (Colquitt et al., 2021). In the canary (*Serinus canaria*), astrocyte density and complexity in HVC correlate with reproductive behaviors (Kafitz et al., 1999; Nottebohm et al., 1986). Astrocyte abundance is also associated with brain size and cognitive ability across species (Colombo et al., 2006; Diamond et al., 1985; Eroglu et al., 2009; Oberheim et al., 2009; Stogsdill and Eroglu, 2017).

Beyond telencephalic song nuclei, astrocytes are abundant in white matter regions like the pallidum and the Fasciculus Prosencephali Lateralis (FPL), which is involved in song production, territoriality, and mating behavior (Bradbury and Vehrencamp, 2011; Coenen et al., 2018; Hamaide et al., 2020; Hernandez et al., 2006; Reiner et al., 2004). These circuits connect the hypothalamus, limbic system, and basal ganglia, suggesting that astrocytes could facilitate early developmental connectivity and vocal motor coordination (Colquitt et al., 2021; Kafitz et al., 1999; Oberheim et al., 2009; Turk et al., 2021).

We propose that astrocytes are key contributors to volitional vocal behaviors, including human speech and birdsong. Given the importance of astrocytes in controlling complex motor outputs and their variation across species, we hypothesized that differences in astrocyte morphology and organization reflect the species-specific demands of vocal communication. Therefore, our study aims to compare the distribution and structural complexity of GFAP-positive astrocytes in vocal production regions of humans and Southern house wrens.

## Materials and methods

### Human brain samples

Postmortem brain tissue from five healthy human donors was obtained with approval from the Bioethical Committee for Human Studies at the University of Antioquia (Record 119, August 2018). Samples included key vocal brain regions involved in speech production and acquisition—Broca’s area, the Laryngeal Motor Cortex (LMC), Sensorimotor cortex (SMC), Wernicke’s area, and the Striatum. Neuropathological classification followed established criteria: CERAD, Braak staging, Thal phase, and NIA-AA guidelines. Donor demographics and clinical data are summarized in Table 1.

**TABLE 1:** Human brain samples used in the study.

Condition	Age of death	Gender	P.I. (h)	CERAD	Braak	Thal	NIA-AA
H1	38	M	3, 4	0	1	2	A1, B1, C1
H2	62	M	24, 9	0	0	0	A0, B0, C0
H3	56	F	5, 6	0	0	0	A0, B0, C0
H4	70	M	3, 6	0	0	0	A0, B0, C0
H5	56	M	11, 0	B	1	2	A1, B1, C2

Demographic and clinical information of human donor samples, including sex, postmortem interval (PI), and neuropathological classifications. M, Male; F, Female.

### Birdsong samples

Southern house wrens (*Troglodytes musculus*, *n* = 5) were collected as part of an authorized scientific permit (ANLA resolution 1,461, December 3, 2014) and with ethical approval (Record 139, March 2021). This species, considered abundant and widespread in urban and agricultural settings, has a conservation status of “least concern” (Johnson, 1998). All procedures complied with the ARRIVE guidelines, NIH’s Guide for the Care and Use of Laboratory Animals (8th edition), and Colombian animal welfare regulations (law 84/1989 and resolution 8,430/1993), with final approval by the University of Antioquia’s Ethics Committee for Animal Experimentation. Birds were classified as juvenile or adult using combined behavioral and anatomical indicators. During mist-net capture, all individuals responded aggressively to conspecific playback-territorial and reproductive behaviors consistent with adult activity. In addition, only individuals that produced clear, structured songs during field monitoring were included, ensuring that sampled birds were actively singing and displaying typical adult vocal behavior during the reproductive season. Adult status was further confirmed by gonadal size and development. Breast, liver, and metatarsus tissues were examined for pathological or hormonal abnormalities, with none detected. All birds were collected in the same rural–semi-urban locality where the species naturally coexists with humans, ensuring comparable environmental conditions. Although Colombia lacks sharply defined seasons, sampling occurred during the species’ breeding period (October-March), which spans both rainy and dry phases. Detailed information for each individual is provided in Table 2.

**TABLE 2:** Morphometric data of Southern house wrens used in the study.

Individual	Sex	Exposed culmen (mm)	Total culmen (mm)	Beak width (mm)	Beak height (mm)	Tarsus length (mm)	Closed Wing Chord (mm)	Weight (g)
Ta1	Male	14.4	10.2	4.5	4.7	13.1	60	16.70
Ta2	Male	12.7	16.6	4.8	4.0	–	60	15.98
Ta3	Male	13.5	15.7	5.7	4.0	23.5	55	15.33
Ta4	Male	12.1	18.1	4.5	3.9	13.4	55	15.29
Ta5	Male	12.4	17.5	4.7	3.8	21.7	56	–

Collection details and morphometric measurements of the five *Troglodytes musculus* individuals. Ta1–Ta5, Southern house wrens 1–5.

### Vocal circuits area in human and songbird isolation and processing

Formalin-fixed human brains (4%) were provided by the Neurobank of the Grupo de Neurociencias de Antioquia. Brain regions involved in speech production and memory (Wernicke’s, Broca’s, LMC, SMC, and Striatum) were dissected and transferred to 4% paraformaldehyde (PFA) in cytoskeleton buffer, changed every 24 h for 3 days.

Southern house wrens were captured using mist nets (12 × 2 m, 30 mm mesh) between 6:00 and 13:00 h following biodiversity inventory protocols. After morphometric measurements, birds were anesthetized with isoflurane (2 mL/125 g) and euthanized by decapitation. Brains were extracted, rinsed in 1 × phosphate-buffered saline (PBS), and fixed in 4% paraformaldehyde (PFA) prepared in CBS buffer (MES 1 M, Sigma-Aldrich M3671; MgCl2 0.3 M, Sigma-Aldrich 814733; EGTA 0.2 M, Millipore 324626; KCl 138 mM, Supelco 104938; KOH pearls). Tissue fixation was performed for 24 h on three consecutive days (24 h × 3 days). After fixation, a sucrose gradient (7, 25, and 30%) was applied over 3 days, increasing the concentration daily until tissues were fully cryoprotected.

### Tissue sectioning and Nissl staining

After cryoprotection, bird brains were washed in 0.1 M phosphate buffer (PB, pH 7.4), hemisected, and sectioned sagittally at –20°C using a Leica CM1850 UV cryostat. Tissues were embedded in OCT and frozen for 3–5 min. Uniform slicing at 30 μm thickness was performed, changing the blade every 20 cuts. Sections were stored in PB with 0.1% azide or cryopreservative. Human brain tissues were processed similarly, sectioned at 50 μm, and stored in 12-well plates with cryopreservative solution.

Sections were washed with 0.1 M phosphate buffer (PB), mounted on charged slides (CAT 22-037-246), and air-dried. After rehydration with distilled water, toluidine blue (1:30) was applied for 6 min. Tissues were dehydrated through 70, 96, and 100% ethanol, cleared with xylene, and mounted with Shandon Consul-Mount (Thermo Scientific; 9990440).

### Immunohistochemistry

For antigen retrieval, human tissues were incubated in 85% formic acid at 85°C for 8 min, followed by incubation in phosphate buffer (PB). Bird sections were treated with citrate buffer (pH 6) (Master-Diagnostic; MAD-004071R/D) at 95°C for 20 min. Peroxidase activity was blocked with a commercial solution (Master-Diagnostic; MAD-004071R/D). Tissues were permeabilized with 0.3% Triton X-100 (Sigma-Aldrich, T9284-500ML) in phosphate buffer and preincubated in 1% bovine serum albumin (BSA; Sigma-Aldrich, A9647), 0.3% Triton X-100, and 0.1 M PB (pH 7.4) for 1 h at room temperature with constant agitation.

Sections were incubated for 72 h at 4°C with primary antibodies: rabbit anti-GFAP (Agilent; Z0334—RRID:AB_10013382; 1:500) for human tissue, and mouse anti-GFAP (Sigma-Aldrich; G3893—RRID:AB_477010; 1:250) for bird samples. After washing, biotinylated goat anti-rabbit (Invitrogen; B-2770—RRID:AB_2536431; 1:250) and goat anti-mouse (Invitrogen; 31800—RRID:AB_228305; 1:250) secondary antibodies were applied for 2 h in a solution containing 0.3% BSA, 0.3% Triton X-100, and 0.1 M PB.

Peroxidase signal was developed using the Avidin–Biotin Complex (ABC) peroxidase staining kit (Thermo Scientific; 32020) and 3,3’-diaminobenzidine (DAB) tablets (Sigma-Aldrich; D4293). Sections were dehydrated through an ethanol gradient (70, 96, 100%) followed by xylene and mounted on charged slides. Finally, coverslips were applied using Consul-Mount (Thermo Scientific; 9990440).

### Tissue scanning and image analysis

Nissl-stained human samples were scanned in 11 Z-layers and GFAP immunostained bird samples in one Z-layer using Ventana DP200 slide scanner (Roche), 20X objective (NA 0.75; UPlanSApo; Olympus) and using 40x digital zoom. Three random visual fields of 2 mm^2^ in area for GM and WM from each slide were selected, processed, and analyzed using QuPath 0.3.2. Cell density quantification was performed using QuPath 0.4.0 in defined ROIs (1,150 × 2,750 μm) by the plugin “positive cell detection.”

GFAP and GS distribution analysis was conducted using FIJI (Hamaide et al., 2020), with signal intensity profiles obtained from cortical (human) and telencephalic/midbrain (bird) regions.

### Bright-field microscope imaging of human and birdsong astrocytes

Five astrocytes per region were randomly imaged (25 total images per region) per species. Bird telencephalic astrocytes (LEP, vascular pallium, and FPL) were visualized using an Olympus CX35 microscope, (Model X31RBSFA) equipped with a 100X oil immersion objective (NA 1.25; PlanC N; Olympus) and 40X objective (NA 0.65; PlanC N; Olympus) with Swift SC1803R camera. Human astrocytes were imaged in layer V of the cortex, and gray matter in Striatum using an Olympus EP50 system.

### Immunofluorescence

Human antigen retrieval was carried out with 85% formic acid at 85°C (8 min) and citrate buffer (pH 6) (Master-diagnostic; MAD-004071R/D) at 95°C for 20 min for birds. Autofluorescence was blocked with 50 mM ammonium chloride NH4Cl (J.T. Baker; 0660-19) prepared in distillated water for 15 min and Sudan black (Sigma-Aldrich; 199664) for 5 min. After blocking with a solution containing 1% (BSA; Sigma-Aldrich, A9647), 0.3% Triton-X100 (Sigma-Aldrich, T9284-500ML), and 0.1 M PB (pH: 7.4) for 1 h at room temperature with constant agitation, sections were incubated for 72 h with primary antibodies: rabbit anti-GFAP [Agilent; Z0334—RRID:AB_10013382 (1:500)], anti- Glutamine Synthetase GS [Invitrogen; 7H9L16—RRID:AB_2633045 (1:50)] with mouse anti-GFAP [Sigma-Aldrich; G3893—RRID:AB_477010; (1:250)] for humans, rabbit anti-GFAP [Sigma-Aldrich; ab5804—RRID:AB_305124; (1:250)] with mouse anti-NeuN [Sigma-Aldrich; MAB377—RRID:AB_2298772; (1:250)], mouse anti-GFAP [Sigma-Aldrich; G3893—RRID:AB_477010; (1:250)] with anti- Glutamine Synthetase GS [Invitrogen; 7H9L16—RRID:AB_2633045 (1:50)] for birds. Secondary antibodies included Alexa Fluor 488 (Invitrogen; A-11001; 1:750) and Alexa Fluor 594 (Invitrogen; A-11012; 1:750), or Alexa 488 Dk anti Ms [Invitrogen; A32766—RRID:AB_2866493 (1:500)] with Alexa 594 DK anti Rb [Invitrogen; A21207—RRID:AB_141637 (1:500)];followed by nuclear labeling with Hoechst (Invitrogen; H3569, 1:5,000). Subsequently, the samples were vigorously washed in PB (0.1 M) three times and mounted using FluorSave reagent (Millipore; 345789).

### Confocal imaging

Astrocytes in human GM layer V and in the telencephalon of birdsong species were captured by confocal microscopy. Three high-magnification images per slide were obtained from double immunostaining (15 individual astrocytes per region) and acquired using an Olympus FV1000 confocal scanning microscope. Tiled images for each human area were taken in a 1 × 4 matrix using a 40X oil-immersion objective (NA 1.30; UPlanFL N; Olympus), and individual astrocytes were imaged with a 60X oil-immersion objective (NA 1.42; PLAPON; Olympus) with a zoom value of 2.

In the case of songbirds, a single tiled image was obtained in a matrix of 8 × 6, and 1 × 4 tiled images in the pallium for each bird were imaged with a 10X objective (NA 0.40; UPlanSApo; Olympus). Birdsong areas were imaged in Z with a 40X oil-immersion objective (NA 1.30; UPlanSApo; Olympus) and a 60X oil-immersion objective (NA 1.42; PLAPON; Olympus), with a zoom value of 2 for individual astrocytes. Three lasers (488 nm, 594 nm, and DAPI) and FluoView 3.1.1.9 software (Olympus) were used. Sixteen-bit TIFF images of 1,024 × 1,024 pixels (105.47 × 105.47 μm) were obtained with an XY pixel size of 103 and 300 nm between Z-sections.

### Image processing and astrocyte morphometry

Confocal images were deconvolved, processed, segmented and 3D reconstructions were created. The images were deconvolved in Huygens Professional 23.3 software (Scientific Volume Imaging B.V.). Images were deconvolved using the classic maximum likelihood estimation (CMLE) algorithm with a signal-to-noise ratio of 21, and these were deconvolved using the wizard deconvolution with a maximum of 60 iterations. The images were transformed to 8 bits and subsequently processed and analyzed in FIJI software. Immunofluorescence signals were segmented using intensity thresholding by the Huang or Triangle algorithm to standardize the fluorescence signals from all the images. Z projections by the standard deviation of the segmented stacks were used to evaluate astrocyte structure. These Z projections were segmented and used to quantify the astrocyte processes. Last, for illustrative purposes, Z projections and rendering of the deconvolved images were made using the max intensity option.

Astrocyte processes were counted using deconvoluted images. Background illumination was reduced by subtracting a value of 25 using the Image-Pro Plus Subtract tool. FIJI software (NIH ImageJ) was used to process, segment, and analyze the images (Schindelin et al., 2012). For counting the crossing number of astrocyte processes, the images were converted to 8-bits and segmented using intensity thresholding by the Huang algorithm. Skeletonization was performed, within the Simple Neurite Tracer (SNT) plugin, with the *analyze sholl from image* option selected radius was set at 5 μm, measured from the center to the furthest process length. Finally, the images were processed to obtain representative figures.

The cell density quantification of GS-positive astrocytes was performed using 40X human tiled images (Broca, LMC, SMC, Wernicke, and Striatum) and 40X images for songbirds in each area (RA, HVC, LMAN, and Area X), using the software QuPath 0.6.0. The “Cell detection” plugin was used with the following parameters: background radius = 8; sigma = 6; minimum area = 50; maximum area = 1,000; threshold = 50; and cell expansion = 0. Nonspecific detections were manually removed when they did not correspond to an astrocyte.

### Orthologous genes

Astrocytes markers sequences from humans (*Homo sapiens*) and songbirds, specifically *Serinus canaria* and *Taeniopygia guttata*, the latter representing the closest available annotated genome to *Troglodytes*, were retrieved from the NCBI database. Pairwise alignments were performed using the BLASTp (Basic Local Alignment Search Tool) algorithm on the NCBI platform using default parameters. The resulting identity, similarity, and query coverage percentages were used to assess evolutionary conservation of these astrocytic markers across species.

For GFAP, the sequences analyzed were NP_002046.1 (*Homo sapiens*) and XP_050841796.1 (*Serinus canaria*). For GS, the sequences included NP_001028216.1 (*H. sapiens*), XP_030082364.1 (*S. canaria*), and XP_002196326.1 (*T. guttata*). For S100β, the sequences used were NP_006263.1 (*H. sapiens*), XP_050843573.1 (*S. canaria*), and NP_001232389.1 (*T. guttata*). For AQP4, the sequences analyzed were NP_001641.1 (*H. sapiens*), XP_009100894.2 (*S. canaria*), and XP_002196027.6 (*T. guttata*).

### Volumetric analysis

The volumes of three human brains were provided by the Neurobank of the Neuroscience Group of Antioquia. The mean of these volumes was divided by body weight to calculate the brain volume/body weight ratio. For *Troglodites musculus*, we previously reported brain volume using Neurolucida Explorer software (MBF Bioscience—MicroBrightField, Version 2021), and the corresponding brain volume/body weight ratio was calculated for each individual and then averaged.

### sc-RNAseq multi-dataset integration and dataset selection

To perform cross-species integrative analyses, we incorporated publicly available single-cell RNA-seq datasets from humans and songbirds. Human cortical astrocytes were obtained from frontal cortex samples (*n* = 6) (GEO: GSE222494) (Almeida et al., 2024), and human corpus striatum [Putamen (*n* = 6) and Caudate (*n* = 8)] (GEO: GSE152058) (Matsushima et al., 2023). Only control samples without neurological disease were included to establish baseline transcriptional profiles. For songbirds, we included single-cell RNA-seq datasets from two phylogenetically related vocal-learning songbirds: the Bengalese finch (*n* = 2) (*Lonchura striata domestica*, GEO: GSE150486) and the zebra finch (*n* = 3) (GEO: GSE153665) (Colquitt et al., 2021). Both species possess well characterized neural song control circuits, providing established models for vocal learning and sensorimotor integration.

### Preprocessing, quality control, and integration

To enable unified cross-regional analysis while preserving anatomical context, we integrated multiple brain regions within each species into single expression matrices. For humans, prefrontal cortex and corpus striatum (Putamen and Caudate) datasets were combined with regional identity retained as metadata labels. For songbirds, HVC, RA, and Area X from two species (Bengalese finch and zebra finch) were integrated with both species and region annotations preserved. This approach enabled visualization of each species’ complete cellular diversity in unified UMAP projections while maintaining regional distinctions for downstream analyses.

Both datasets underwent identical preprocessing using Scanpy v0.7.4 (Wolf et al., 2018). Doublets were removed with Scrublet v0.2.2 (Wolock et al., 2019). Quality control excluded cells with < 300 or > 6,000 genes (human) or > 3,000 genes (avian), and > 5% mitochondrial content. Genes detected in < 100 cells or with < 5 total counts were filtered. Mitochondrial genes and *MALAT1* were excluded. Counts were normalized to CPM and log-transformed (log1p). Harmony v0.1.0 (Korsunsky et al., 2019) corrected batch effects using brain region and dataset as covariates (50 PCs). Highly variable genes (4,339 human; 2,752 avian) were identified via Seurat v3. PCA retained components capturing 90% variance (13 PCs human; 35 PCs avian). K-NN graphs (*k* = 500 human; *k* = 300 avian) and UMAP embeddings were generated for visualization.

### Clustering and cell-type annotation

Cell populations were identified using Leiden clustering with resolution parameters optimized per species: *r* = 0.2 for humans and *r* = 0.3 for songbirds, selected to balance subtype resolution with biological interpretability. Cell type annotation employed a multi-tiered approach integrating: (i) expression of canonical marker genes, (ii) cross-referencing with curated databases including CellMarker 2.0, Tabula Sapiens, PanglaoDB, and Azimuth, (iii) differential expression analysis to identify cluster-top genes (Wilcoxon rank-sum test, Bonferroni-corrected *p* < 0.05), and (iv) validation against cell-type-specific markers reported in primary literature. This combined strategy ensured robust, literature-supported annotations.

For human datasets, annotated cell types included: medium spiny neurons (MSN-D1: DRD1^+^; MSN-D2: *DRD2*^+^), inhibitory interneurons (*GAD1/2*^+^), excitatory neurons (*CUX2*^+^/*SLC17A7*^+^), astrocytes (*SLC1A2^+^/AQP4*^+^), microglia (*P2RY12^+^/CX3CR1*^+^), oligodendrocytes (*MBP^+^/PLP1*^+^), oligodendrocyte precursor cells (OPC) (*PDGFRA*^+^), vascular cells (*VWF^+^/NOTCH3^+^*).

For avian datasets, cell types included: excitatory neurons (*CUX2^+^/SLC17A7*^+^), inhibitory neurons (*GAD1/2*^+^), astrocytes (*SLC1A2^+^/GFAP*^+^), microglia (*P2RY12*^+^), oligodendrocytes (MBP^+^), oligodendrocyte precursor cells (PDGFRA^+^), vascular cells (VWF^+^), and immature neurons (*SOX4^+^/DCX*^+^). All neurons expressed pan-neuronal marker MAP2. Layer-specific excitatory neuron subtypes (*L2/3: CUX2^+^; L4: RORB^+^; L5: THEMIS^+^; L5/6: SEMA3A*^+^) and interneuron developmental origins (*CGE: ADARB2^+^; MGE: LHX6*^+^) were identified where applicable.

### High-resolution astrocyte subtype analysis and orthology mapping

Astrocyte heterogeneity was investigated through dedicated subclustering of all astrocyte-annotated cells isolated from the integrated multiregional human-songbird dataset. To maximize detection of subtle transcriptional differences within this cell class, astrocytes underwent independent re-processing: raw counts were re-normalized to CPM and log-transformed, highly variable genes (*n* = 2,000) were re-identified using the Seurat v3 method, and principal component analysis was performed retaining components capturing 90% of variance. Harmony v0.1.0 was re-applied for batch correction using species, dataset, and anatomical region as covariates. A k-nearest neighbor graph (*k* = 50) was constructed, and Leiden clustering (resolution = 0.2 for both humans and songbirds) identified transcriptionally distinct astrocyte populations. For each astrocyte subcluster, marker genes were identified using Wilcoxon rank-sum tests comparing each cluster against all others, with significance defined as Bonferroni-corrected *p* < 0.05 and log2 fold-change > 0.5. Comparative visualization employed dot plots with dual-parameter encoding: dot color represents mean normalized expression calculated as the average log1 *p*-value across all cells in each subcluster, scaled from 0 (no expression) to maximum expression across clusters; dot size indicates percentage of cells expressing the gene within each subcluster (detection threshold > 0 raw counts), ranging from 0% (no dot) to 100% (maximum size). Genes and clusters were ordered to reveal conserved molecular programs and species-specific astrocyte signatures.

### Statistical analysis

The sample size for each experimental group was defined as each individual (*n* = 5 for humans and birds), and each individual was analyzed as an independent experiment. Each dot in the plots represents one individual, and its value corresponds to the average of at least five regions of interest (ROIs) per speech-related area in humans and song-related area in birds. In each ROI, we quantified either astrocyte number or the morphology-based complexity parameters from a minimum of 10 astrocytes. Normality was assessed using the Shapiro-Wilk test. Parametric univariate data were also analyzed using one-way ANOVA and Tukey’s multiple comparison tests. The non-parametric data will be analyzed with the Kruskal–Wallis test and Dunn’s test. Student’s *t*-test was used in comparative analysis between species. Two-way ANOVA was used for compared Sholl analysis. All groups were processed in parallel to reduce between-trial variation. Analyses were performed using GraphPad Prism version 8.0 software. Results were considered significant at *p < 0.05, **p < 0.01 and ***p < 0.001.

## Results

### Differential cellular density in the vocal areas of humans and southern house wren

**FIGURE 1:**
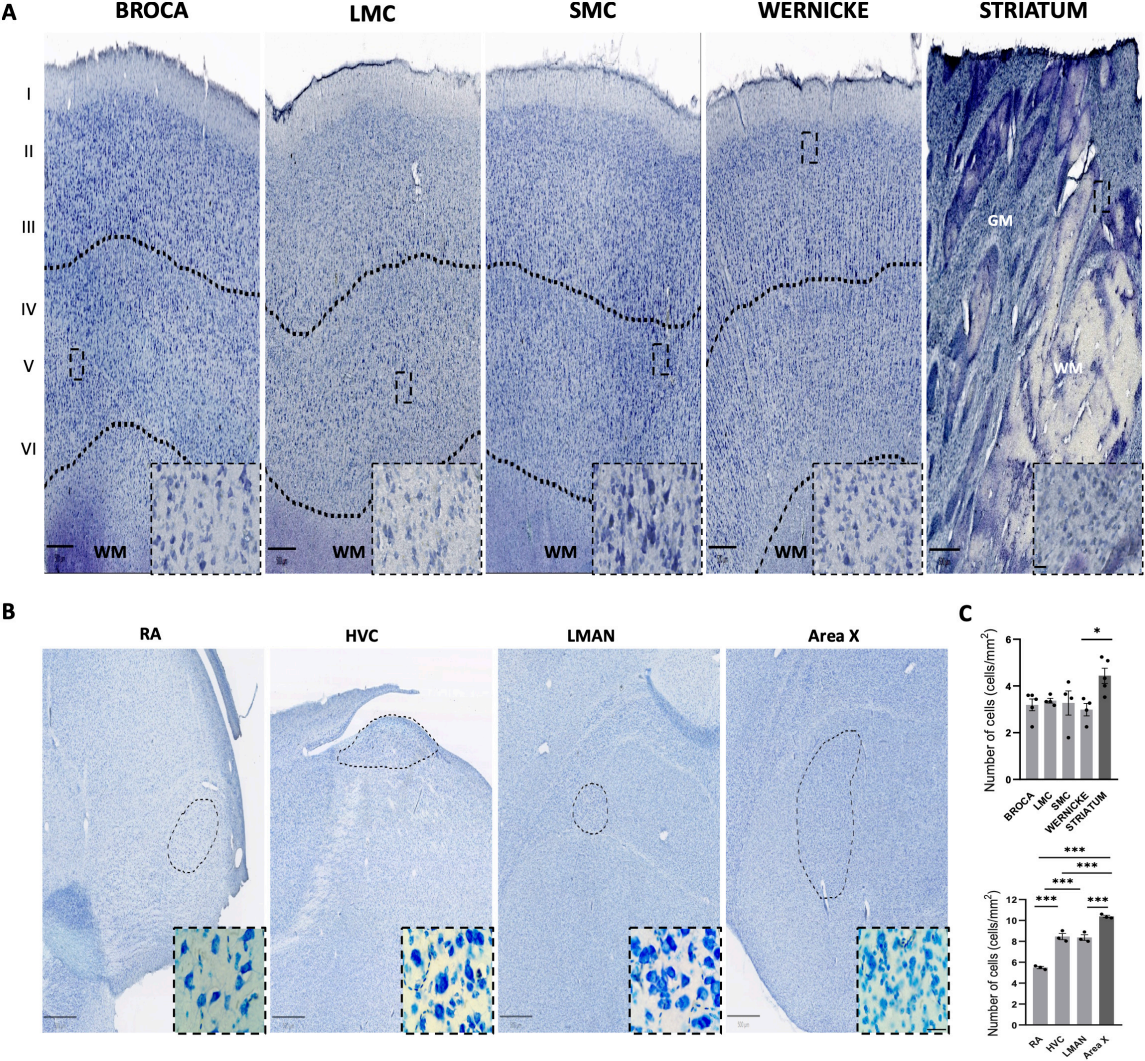
Areas involved in speech production and acquisition in humans and song in birds. **(A)** Laminar representation of Nissl staining (20x-objective scanned slices) from human brain cortex regions associated with production (Broca’s area, Laryngeal Motor Cortex (LMC), and Sensorimotor Cortex (SMC) and acquisition/memory (Wernicke’s area and Striatum), with corresponding zoomed crops. The upper dashed line marks the boundary of layer III (upper layers I–III), and the bottom dashed line indicates the boundary between gray and white matter. **(B)** Bright field images of Southern house wren brain areas involved in song production [HVC, Robust nucleus of arcopallium (RA)] and acquisition/memory [(Area X, Lateral magnocellular nucleus of the anterior nidopallium (LMAN), HVC)]. Scale bars: 500 μm (whole brain images), 20 μm (40x human magnified crops), 25 μm (Southern house wren 100x-objective images). **(C)** Graphs of the quantitative analysis of cell number/area in speech- and song-related areas in human (up) and Southern house wren (down), respectively. Humans shows a significant difference between Wernicke and Striatum, “*p* = 0.0337.” In Southern house wren, we show several significant differences between the areas: in HVC vs. RA (“*p <* 0.0001”); RA-LMAN (“*p <* 0.0001”); RA-Area X (“*p* < 0.0001”); HVC-Area X (“*p* = 0.0010”) and between LMAN-Area X (“*p* = 0.0007”). Striatum in humans and Area X in the Southern house wren exhibit the highest cellularity. Statistical analyses were performed using one-way ANOVA with Tukey’s multiple comparisons test. Each dot represents the mean value per individual. *p < 0.05, ***p < 0.001. Bars are presented as mean ± SEM.

We analyzed postmortem brain tissue from healthy human donors without neurological disease (confirmed by CERAD, Braak, Thal, and NIA-AA scores) aged 38–70 years (Table 1), and adult male Southern house wrens, selected based on morphometric criteria (Table 2; Johnson, 1998). A neuroanatomical comparison was performed to assess cellular density in regions involved in the acquisition, memory, and production of speech in humans and song in birds (Figure 1A).

In humans, we delineated the laminar organization of the cerebral cortex in gray matter (GM) from Broca’s area, Wernicke’s area, LMC, and SMC, dividing them into upper (I–III) and deeper (IV–VI) layers, as well as white matter (WM). In the striatum, we analyzed cellular distribution within WM.

For the Southern house wren, we referenced our previously developed vocal brain atlas (López-Murillo et al., 2024), identifying mediolateral coordinates for LMAN (180–1,650 μm), Area X (270–1,530 μm), RA (270–1,170 μm), and HVC (390–2,760 μm) (Supplementary Figure 1). We described the nuclear organization of these regions, which are responsible for song learning, memory, and production (Figure 1B).

No significant differences were observed in average cell density between the upper and deeper cortical layers in humans (Figures 1A,C). However, the striatum exhibited notably higher cell density. In birds, Area X displayed the highest cell density, followed by HVC and LMAN, with RA showing the lowest (Figures 1B,C). These data suggest the basal ganglia may play a central role in vocal control in both species.

### Basal ganglia GFAP astrocytes are more complex in humans and southern house wren vocal areas

We analyzed the distribution of GFAP-positive astrocytes in vocal brain regions of humans and Southern house wrens using the same anti-GFAP antibody. Previous findings confirmed that this antibody effectively detects GFAP in humans and Southern house wrens but not in tailed-rufous hummingbirds (López-Murillo et al., 2024), supporting GFAP antigen homology between humans and songbirds.

**FIGURE 2:**
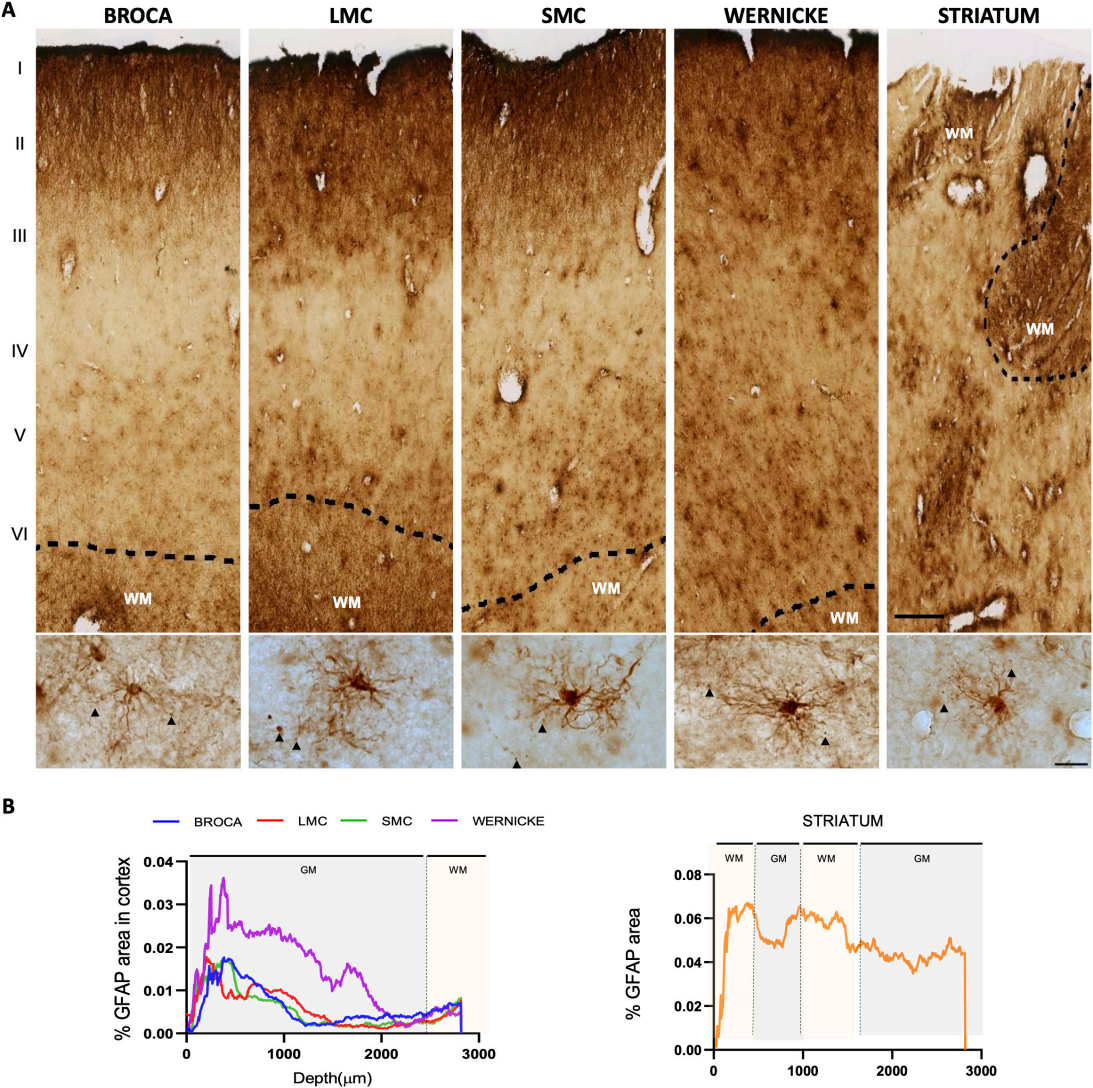
Distribution of GFAP-positive astrocytes in human speech-related areas. (**A,** up) Laminar representation of scanned images (20x-objective scanned slices) of Glial fibrillary acidic protein (GFAP)-positive astrocytes across the cerebral cortex; the Striatum does not show a layered organization. (**A**, Down) Bright field images (100x-objective images) of GFAP astrocytes in the layer V of cortex exhibit branching with varicosities (arrowhead inset). Scale bar: 100 μm (laminar representation images); 20 μm (individual 100x-objective astrocytes). (**B**, left) GFAP-positive line profile across cortical layers, showing an increased GFAP in primary cortical layers and in white matter, and reduced GFAP in deeper layers of the cortex. (**B**, right) Line profile in the Striatum indicates elevated GFAP in white matter. Dotted lines mark the boundary between gray and white matter.

In humans, GFAP+ astrocytes were observed in both GM and WM, characterized by elongated processes with varicosities (Figure 2A). Astrocytes were predominantly located in the upper cortical layers (I–III) of Broca’s, Wernicke’s, LMC, and SMC, with reduced presence in deeper layers and a secondary increase in WM (Figure 2B). In the striatum, GFAP+ astrocytes were abundant and diffusely distributed throughout WM.

**FIGURE 3:**
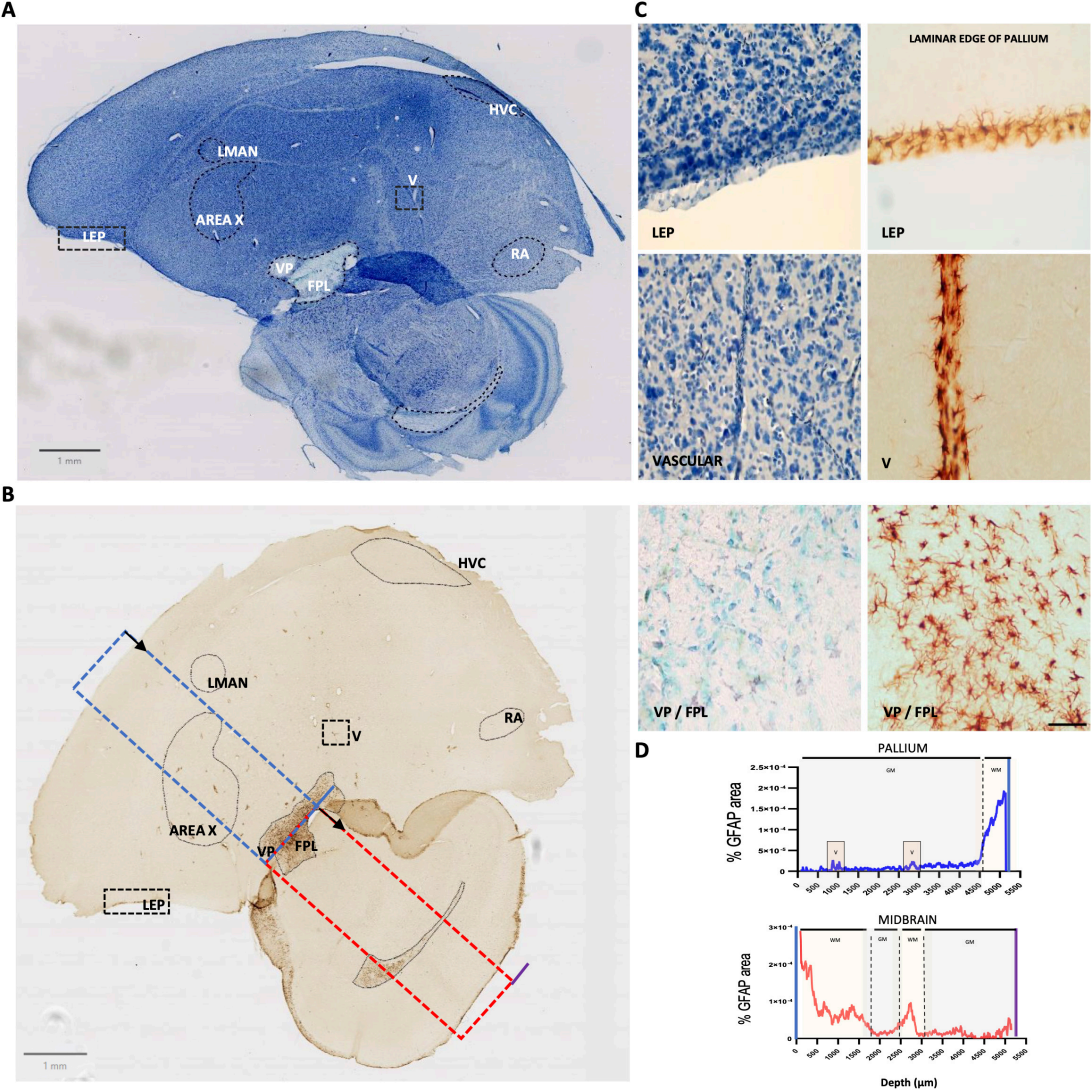
Distribution of GFAP-positive astrocytes in the Southern house wren. **(A)** Nissl staining (scanned 20x-objective) showing the song control nuclei [Robust nucleus of arcopallium (RA), HVC, Lateral magnocellular nucleus of the anterior nidopallium (LMAN), Area X] and other brain areas [Laminar edge of the pallium (LEP)], Ventral pallidum (VP), vasculature, (V) and lateral Fasciculus Prosencephali Lateralis (FPL), with dashed outlines. **(B)** Glial fibrillary acidic protein (GFAP) immunohistochemistry (IHC) (scanned 20x-objective) highlights astrocyte-positive regions. Blue and red dotted rectangles indicate regions used for line profile analysis; black arrows show direction of measurement. **(C)** Bright field images (40X) for Nissl staining (left) and IHC (right) for GFAP-positive regions (LEP, vasculature, VP / FPL). Scale bars: 1 mm (scanned images); 20 μm (40X images). **(D)** Line profiles of GFAP signal in the Pallium (blue) and midbrain (red) reveal increased positive GFAP astrocytes expression in vasculature and white matter.

In Southern house wrens, classical astrocytic morphology was not observed in HVC, RA, LMAN, or Area X when stained with either human or avian-specific anti-GFAP antibodies. It was reproduced with a specific anti-GFAP avian antibody (Figure 3A). However, whole-tissue scans revealed GFAP+ astrocytes in two distinct regions: the pallium [Laminar edge of the pallium (LEP), and vascular areas (V)], and the pallidum, specifically in the Fasciculus Prosencephali Lateralis (FPL) (Figures 3B,C). Like human tissues, a GFAP astrocytes distribution profile was performed in the pallium-to-pallidum, as shown in Figure 3A, where the blue dotted box represents the region of interest analyzed in the telencephalon. Distribution profiles confirmed that astrocytes were mainly localized in WM, particularly in the FPL, while in the pallium they were restricted to vasculature and the laminar edge (Figure 3D). These results suggest a heterogeneous and region-specific distribution of GFAP astrocytes, with enrichment in thalamo-basal ganglia circuits in both species.

**FIGURE 4:**
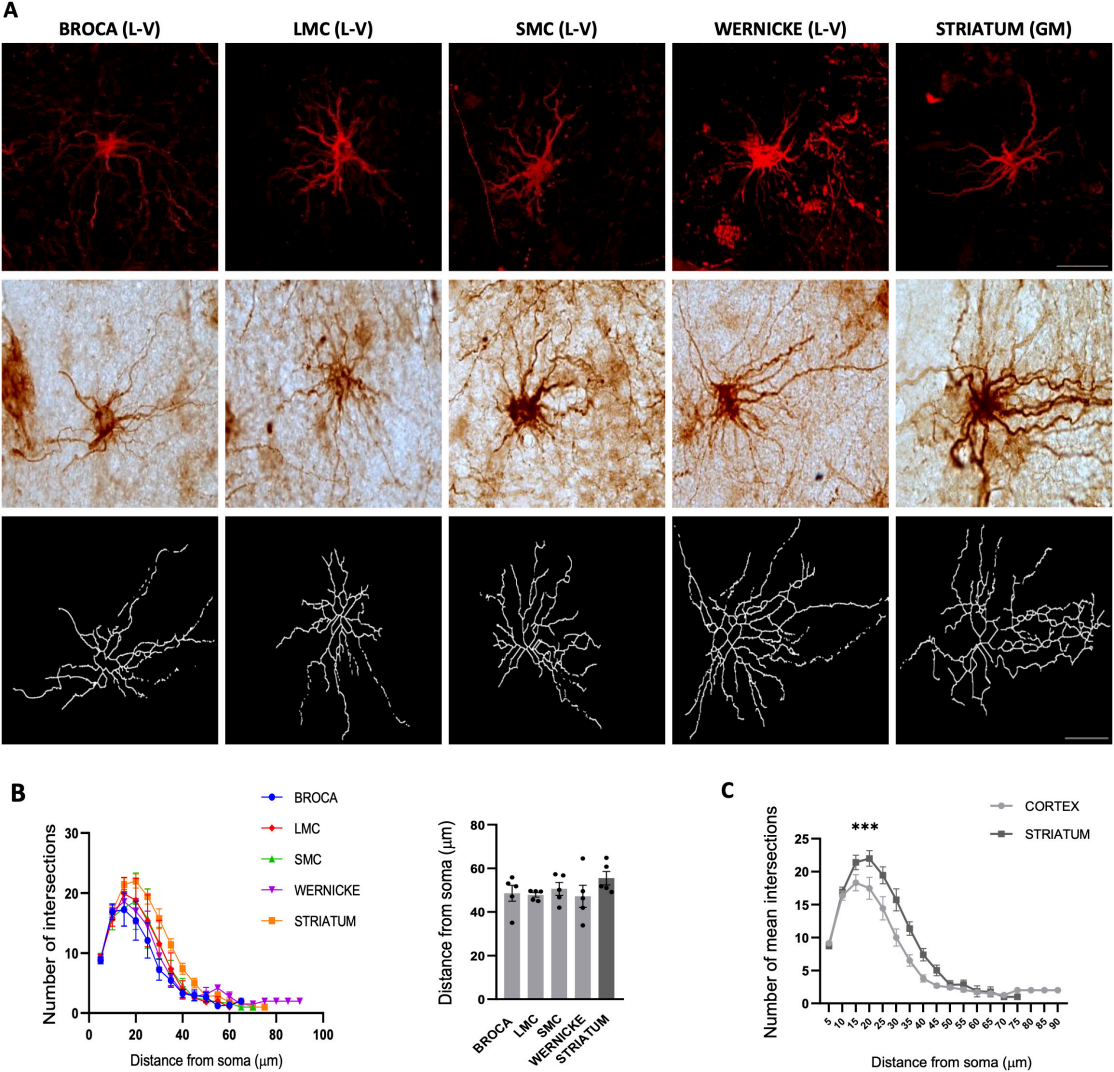
Morphological analysis of GFAP-positive astrocytes in humans. (**A**, top) Representative z-projection confocal images (60X) for GFAP-positive astrocytes in speech-related areas; images captured in layers V in the cortex and gray matter in the striatum. (**A**, middle) Bright field 100x-objective IHC images of Glial fibrillary acidic protein (GFAP)-positive astrocytes in speech-related areas in humans (**A**, bottom) Skeletonized representations of immunohistochemistry (IHC) astrocyte morphology. Scale bars: 25 μm for 60X IF and 100x-objective IHC images. (**B**, left) Sholl analysis shows no significant differences in number of intersections or distance from nucleus of astrocytes in speech-related areas; (**B**, right) Maximum process distance from nucleus is also similar across regions. **(C)** Sholl comparison reveals that striatal astrocytes have significantly more intersections (15–45 μm from soma) than cortical astrocytes. Statistical analysis was performed using Kruskal–Wallis with Dunn’s test and two-way ANOVA for Sholl analysis. The significance levels were set at ***p < 0.001. Bars are presented as mean ± SEM.

Astrocyte heterogeneity between GM and WM is well established (Köhler et al., 2021), with marker-dependent subtypes. To characterize astrocyte morphology in humans, we analyzed GFAP+ astrocytes in layer V of the cortex (Figure 4A), previously shown to have gene expression convergence with RA and HVC in birds (Colquitt et al., 2021). No significant branching differences were observed among cortical regions (Figure 4B). However, striatal astrocytes exhibited significantly more complex branching patterns compared to cortical regions (Figure 4C).

In Southern house wrens, we previously demonstrated that GFAP Astrocytes show a region-specific distribution across the telencephalon, with GFAP expression restricted to the LEP, vascular territories, and the FPL near the basal ganglia (López-Murillo et al., 2024). In that study, LEP and vascular GFAP Astrocytes displayed enlarged somas with few, short processes, whereas astrocytes in the FPL exhibited larger somas and more elongated processes. These findings, reproduced here in Supplementary Figures 2A,B, confirm the increased astrocytic complexity associated with basal ganglia–related regions in the Southern house wren.

### Human GFAP astrocytes exhibit greater branching than those in southern house wren

**FIGURE 5:**
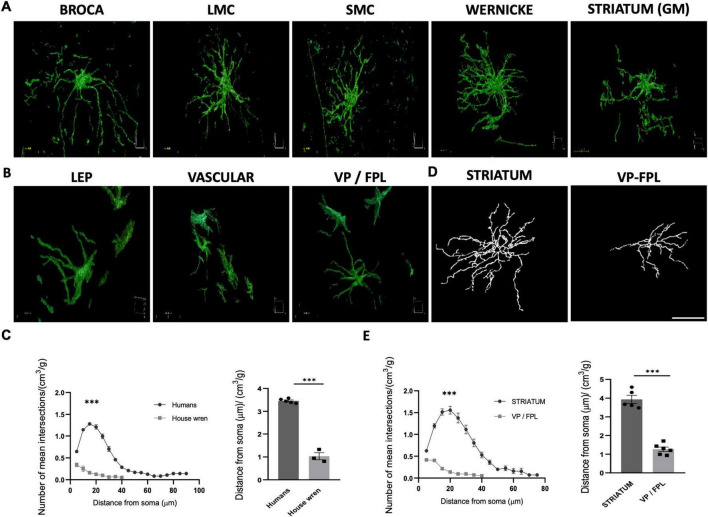
Comparative analysis of human and Southern house wren astrocytes. **(A,B)** 3D reconstructions of representative astrocytes in each species. Scale bar: 25 μm for 60X images. (**C**, left) Sholl analysis comparing astrocyte complexity normalized to brain volume/body mass; (**C**, right) Human astrocytes processes show significant greater distance from soma (“*p* < 0.0001”) than Southern house wren. **(D)** Skeletonized images of astrocytes from the human striatum and wren Ventral Pallidum (VP)/Fasciculus Prosencephali Lateralis (FPL). **(E)** Sholl analysis reveal human striatal astrocytes have significant greater number of intersections and distance from soma (“*p* < 0.001”) than (VP) / (FPL) astrocytes, normalized to brain volume/body mass. Statistical analyses were performed using an Unpaired t test for bar graphs, and two-way ANOVA for Sholl analysis. ***p < 0.001.

To determine differences in the morphology of GFAP astrocytes between humans and wrens, we compared branching and maximum length in both species. We conducted a morphological analysis of GFAP astrocytes using immunofluorescence and confocal microscopy. To assess the allometric relationship of astrocyte complexity relative to brain size, we normalized the average number of intersections and lengths of GFAP astrocytes to the mean brain volume-to-body weight ratio—19.01 mm^3^/g for Southern house wrens and 14.12 mm^3^/g for humans. This analysis provided three-dimensional insights into astrocyte structure (Figures 5A,B). We found that the average number of intersections and the maximum length of GFAP astrocytes from human vocal areas were greater than those from wrens (Figure 5C).

A morphological comparison between human and birdsong astrocytes revealed that astrocytes from both species exhibit morphological heterogeneity depending on GFAP localization. Specifically, a comparison of GFAP astrocytes in the basal ganglia showed the same trend: human astrocytes exhibited greater branching than those in the Southern house wren (Figures 5D,E). Basal ganglia GFAP astrocytes exhibited significantly more branching in humans than in Southern house wrens. These findings suggest that GFAP astrocytes are substantially more complex in humans than in Southern house wrens.

### Broadly uniform GS astrocytes in the southern house wren resembling human astrocytes

To characterize astrocytic subpopulations in the Southern house wren, we first performed a double labeling of S100β and GFAP. S100β reproduced the same restricted pattern previously described for GFAP, showing positive cells at the LEP, within vascular territories, and inside HVC and Area X, where astrocytes were predominantly associated with vasculature. This organization matched the punctate and fibrillar S100β distribution earlier reported in Southern house wrens (López-Murillo et al., 2024) and is illustrated in Supplementary Figure 2C. These findings indicate that S100β and GFAP label the same spatially restricted astrocytic subset within the telencephalon.

**FIGURE 6:**
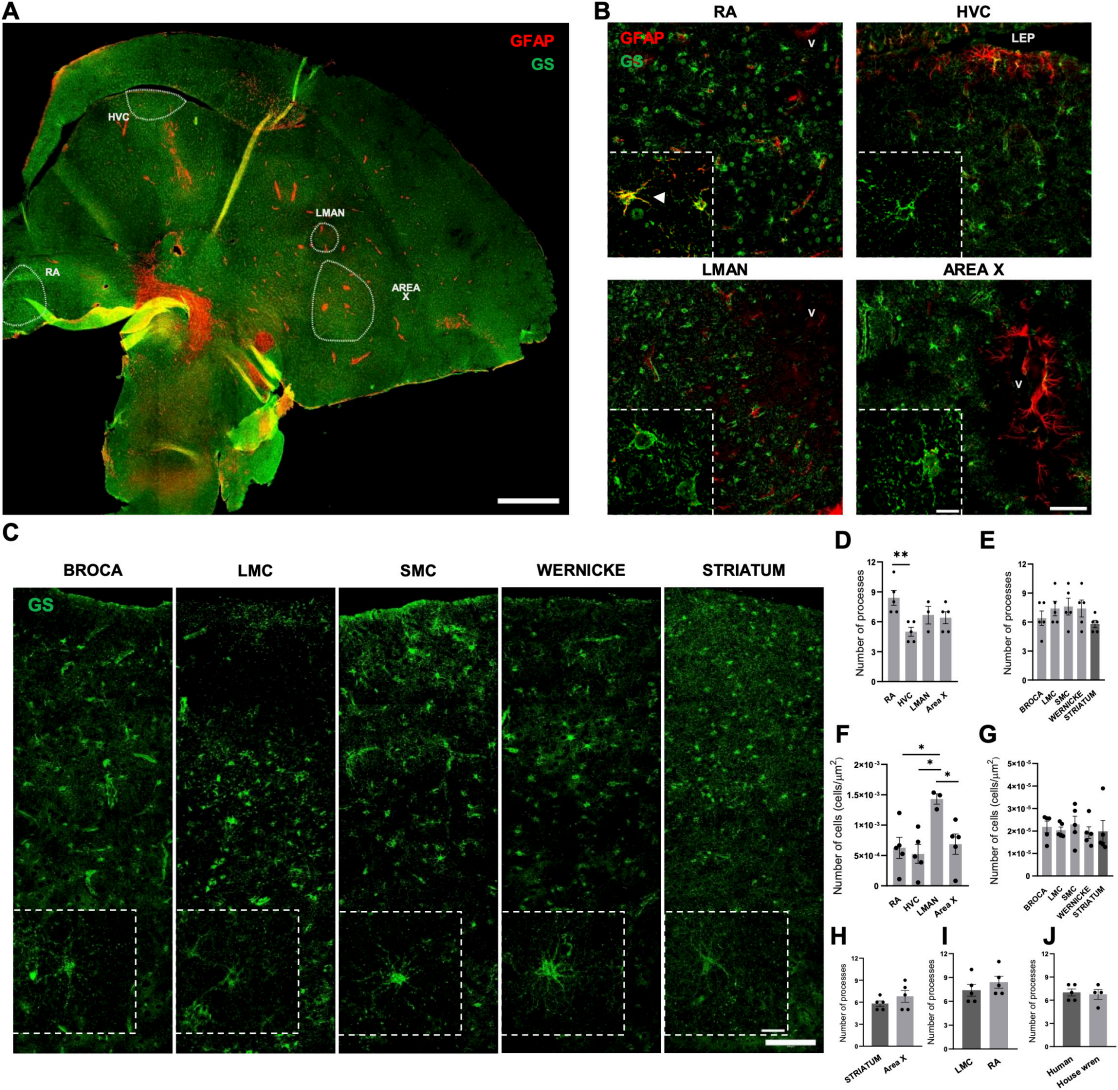
GS Astrocytes reveal a conserved astroglial organization in humans and the Southern house wren. **(A)** Low-magnification stitched projection (40X objective) showing the distribution of Glial fibrillary acidic protein (GFAP) Astrocytes (red) and Glutamine synthetase (GS) Astrocytes (green) across the Southern house wren pallium and pallidum. Dashed contours outline the vocal nuclei Robust nucleus of arcopallium (RA), HVC, Lateral magnocellular nucleus of the anterior nidopallium (LMAN), and Area X. Scale bar: 1 mm. **(B)** Confocal z-projection images (40X) from RA, HVC, LMAN, and Area X in the Southern house wren, with corresponding insets of maximal projection (60X) illustrating individual astrocytes. GFAP Astrocytes appear sparsely and mainly near vascular territories, whereas GS Astrocytes are distributed continuously throughout each nucleus. Arrowhead shows GFAP-positive and GS-positive astrocytes. **(C)** Representative 40X tiled images from human Broca’s area, Laryngeal Motor Cortex LMC, Sensorimotor Cortex (SMC), Wernicke’s area, and the striatum showing respective Z-projections of GS-positive astrocytes (60X). GS labeling in human cortical speech areas demonstrating a continuous laminar distribution from layers I–VI and extending into adjacent white matter. Scale bars: 100 μm (tiled images), 20 μm (60X images). **(D)** Quantification of the number of primary processes per GS Astrocyte in Southern house wren show a significant difference between RA-HVC, “*p* = 0.0082.” One-way ANOVA with Tukey’s multiple comparisons test was used to evaluate regional differences. **(E)** Quantification reveals no significant differences of primary processes per GS Astrocyte in human cortical speech regions One-way ANOVA with Tukey’s multiple comparisons test was used to evaluate differences. **(F)** Quantification of number of GS-positive cells/area in Southern house wren shows significant differences between LMAN and the other regions (LMAN-RA, “*p* = 0.0311”; LMAN-HVC, “*p* = 0.0148”; LMAN-Area X, “*p* = 0.0487”). One-way ANOVA with Tukey’s multiple comparisons test was used to evaluate differences. **(G)** No significant differences were observed in the number of GS-positive cells/area in human speech regions. **(H)** Cross-species comparison of the number of primary processes between human striatal GS Astrocytes and GS Astrocytes from Area X in the Southern house wren, evaluated using unpaired Student’s *t*-test. **(I)** Cross-species comparison between human cortical GS Astrocytes (LMC and RA) shows no differences in the number of primary processes, evaluated using unpaired Student’s *t*-test. **(J)** No differences are observed in the number of processes between all GS astrocytes in humans’ regions and Southern house wren areas, assessed using Student’s *t*-test. Individual data points represent biological replicates. *p < 0.05, **p < 0.01. Bars are presented as mean ± SEM.

We next performed GS and GFAP labeling and observed that GS-positive astrocytes displayed clear astrocytic morphology in both humans and the Southern house wren (Figures 6A–C). Independently of this morphological similarity, we also found that GS shows high sequence conservation across species, with 100% identity and full query coverage between humans and both *S. canaria* and *T. guttata*. GFAP similarly exhibited high homology (99.29% identity, 98% coverage), while AQP4 displayed complete identity (100%) with partial query coverage (69%). In contrast, S100β presented lower identity (84.78%) despite full coverage. Together, these molecular comparisons support the orthology of the major astrocytic markers across humans and songbirds.

Whereas the S100β and GFAP-positive subset remained spatially restricted, GS-positive astrocytes were present continuously and relatively homogeneously throughout the entire pallium and pallidum of the Southern house wren, revealing a broader astroglial population not detected by the other markers (Supplementary Figure 2D). Because of this extensive GS distribution along the pallium, GS-positive astrocytes were also readily observed within all major vocal nuclei, including HVC, RA, LMAN, and Area X; which are embedded within pallial territories (Figure 6B). Although GS-positive astrocytes were widely distributed, a subset displayed a vascular-associated pattern that overlapped with GFAP labeling, a feature that appeared more noticeable in RA but was still present across regions. In these same regions, GFAP-positive astrocytes remained sparse and predominantly associated with vascular structures, with only occasional GFAP-positive processes extending into LMAN and Area X.

In human cortical and striatal speech regions, including Broca’s area, LMC, SMC, Wernicke’s area, and the striatum; GS-positive astrocytes spanned all cortical layers (I–VI) and extended into adjacent WM (Figure 6C). This laminar organization paralleled the homogeneous GS distribution observed in the Southern house wren pallium and aligned with previous descriptions of GS expression in human frontal cortex and control tissue (Supplementary Figure 2D).

Quantification of primary processes (Figure 6D) revealed no regional differences in GS Astrocytes across human cortical or striatal regions. In the Southern house wren, however, GS Astrocytes in RA exhibited a higher number of primary processes compared with HVC, LMAN, and Area X (Figure 6E).

Additionally, when comparing the number of GS-positive astrocytes across regions, humans showed no notable differences among areas. In contrast, the house wren exhibited a higher number of GS-positive astrocytes in LMAN relative to HVC, RA, and Area X (Figures 6F,G).

Cross-species comparisons showed no significant differences between human LMC GS astrocytes and GS astrocytes in RA, nor between human striatal GS Astrocytes and GS Astrocytes in Area X (Figures 6H,I). When values were averaged across all investigated regions, the total number of primary processes was comparable between humans and the Southern house wren (Figure 6J).

In summary, GS Astrocytes revealed a conserved and broadly homogeneous astroglial organization across species, while the GFAP pattern points to a more specialized subset.

### Cross-species single-cell transcriptomic comparison reveals conserved GLUL-expressing astrocytes and distinct GFAP specialization in humans

**FIGURE 7:**
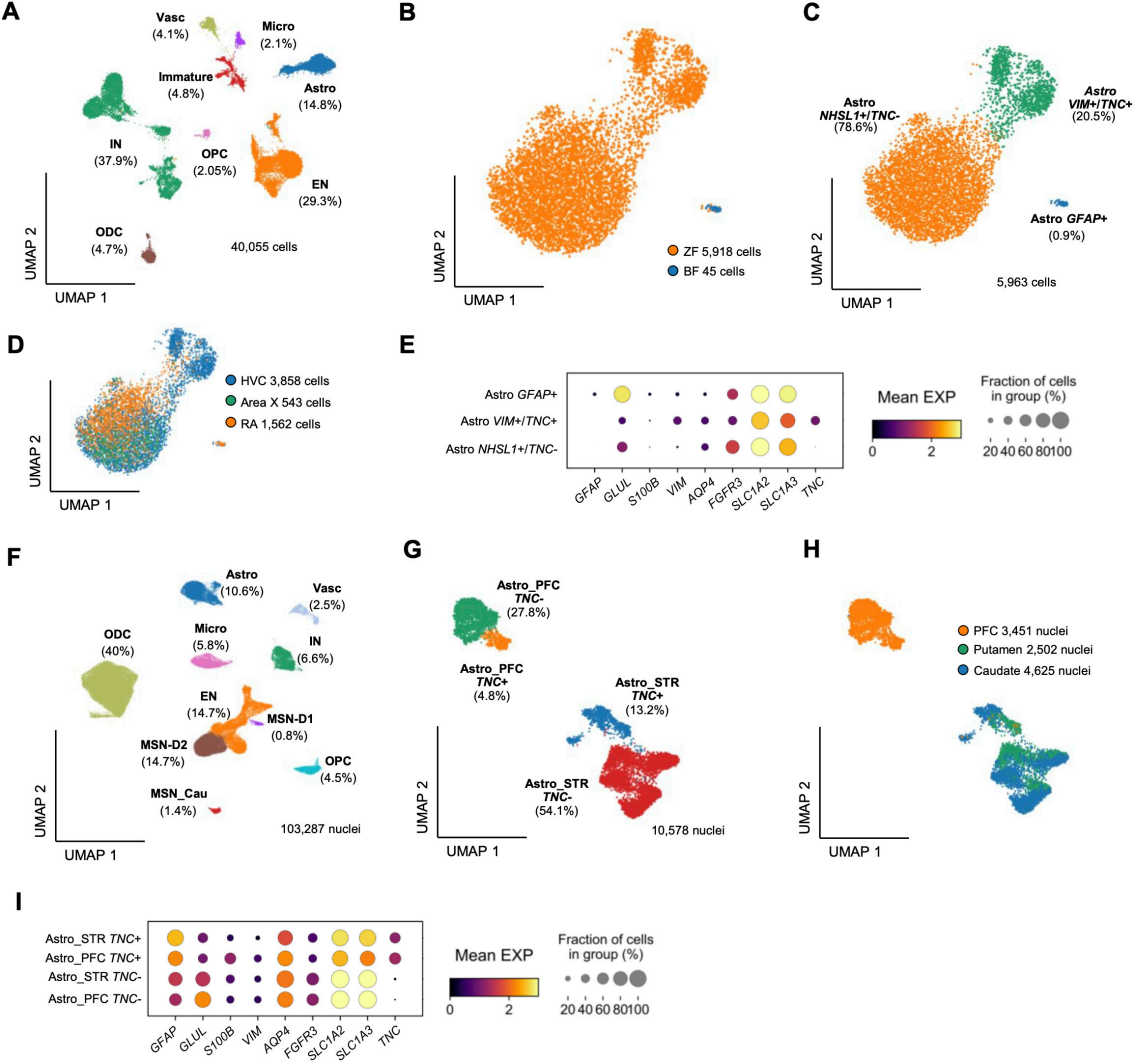
Cross-species single-cell transcriptomic comparison of astrocytes in songbird vocal nuclei and human telencephalon. **(A)** Integrated UMAP of 40,055 avian cells (zebra finch + Bengalese finch) obtained from the vocal nuclei HVC, Robust nucleus of arcopallium (RA), and Area X, showing the major cell classes of the song-control system. **(B)** Astrocyte subclustering from the finch dataset (5,963 astrocytes), where the Bengalese finch contributes only 45 Glial fibrillary acidic protein (GFAP)-positive astrocytes, largely restricted to RA. **(C)** Annotation of zebra finch (ZF) and Bengalese finch (BF) astrocyte subclusters, resolving three transcriptional groups: *NHSL1*^+^/*TNC*^+^ (78.6%), *VIM*^+^/*TNC*^+^ (20.5%), and *GFAP*^+^ (0.9%). **(D)** Contribution of each vocal nucleus to the avian astrocyte dataset: HVC (3,858), Area X (543), and RA (1,562). **(E)** Dot plot of avian astrocyte markers, where circle size reflects the fraction of cells expressing each gene and color indicates mean expression level. **(F)** Integrated UMAP of 103,287 human nuclei from prefrontal cortex, caudate, and putamen, identifying major telencephalic cell classes including three MSN subtypes. **(G)** Subclustering of 10,578 human astrocytes, resolving four groups: PFC–*TNC*^+^ (27.8%), PFC–*TNC*^+^ (4.8%), STR–*TNC*^+^ (54.1%), and STR–*TNC*^+^ (13.2%). **(H)** Regional contribution of human astrocytes: prefrontal cortex (3,451), putamen (2,502), and caudate (4,625). **(I)** Dot plot of human astrocyte markers (*GLUL, GFAP, S100B, AQP4, TNC*), where circle size represents cellular fraction and color denotes mean expression across astrocyte subgroups.

To validate and support our histological findings, we compared astrocyte populations between humans and songbirds through an integrative single-cell RNA-seq analysis using two vocal-learning species, the zebra finch and the Bengalese finch (Colquitt et al., 2021). The avian datasets were obtained from the vocal nuclei HVC, RA, and Area X, capturing the transcriptional landscape of the core song motor–basal ganglia circuit (Figure 7A; Supplementary Figure 3). Subclustering of astrocytes showed that most astroglia originated from the zebra finch, whereas the Bengalese finch contributed a very small subset of GFAP-positive astrocytes, largely restricted to RA (Figure 7B; Supplementary Figure 3). Because the GFAP-positive group represents only 0.9% of all avian astrocytes, this small set of RA-derived Bengalese cells produces an apparent enrichment of RA within this cluster. Astrocytes segregated into three transcriptional subclusters: a *GFAP*-^+^ group, an *NHSL1*^+^ /*TNC*^+^ group, and a *VIM*^+^ / *TNC*^+^ group (Figures 7C,D). Across both finch species, GFAP-positive astrocytes were exceedingly rare, yet the few detected cells displayed relatively high expression. By contrast, *GLUL* (GS) remained robust and widespread across species, aligning with our histological findings (Figure 7E; Supplementary Figures 4A,B).

In humans, integration of prefrontal cortex (PFC) (Almeida et al., 2024) and striatum (Matsushima et al., 2023) datasets identified the major telencephalic cell types (Figure 7F), including three medium spiny neuron (MSN) subtypes characteristic of the basal ganglia. Astrocytes formed four transcriptionally distinct groups that segregated both by region -prefrontal cortex vs. striatum, and by tenascin-C expression (*TNC*^+^ / *TNC*^+^) (Figures 7G,H). Dot-plot visualization revealed that GFAP was broadly and strongly expressed across all astrocytic subclusters, with the highest levels in *TNC*-positive clusters; in contrast, *GLUL* showed uniform expression across the four astrocyte populations, paralleling the strong and homogeneous *GLUL* signal observed in birds (Figure 7I; Supplementary Figures 4C,D). *AQP4* was also widely expressed, whereas *S100B* appeared in all astrocytic groups but at lower levels.

Together, these analyses validate our histological observations by confirming that *GLUL* (GS) is consistently and broadly expressed across astrocytes in both songbird vocal nuclei and human cortical–striatal regions. In contrast, *GFAP* appears only in a small astrocytic subset in birds yet is more widely expressed in human astrocytes, including those from the striatum. These cross-species transcriptomic patterns therefore support the robustness of our GS- and GFAP-based findings in tissue.

## Discussion

**FIGURE 8:**
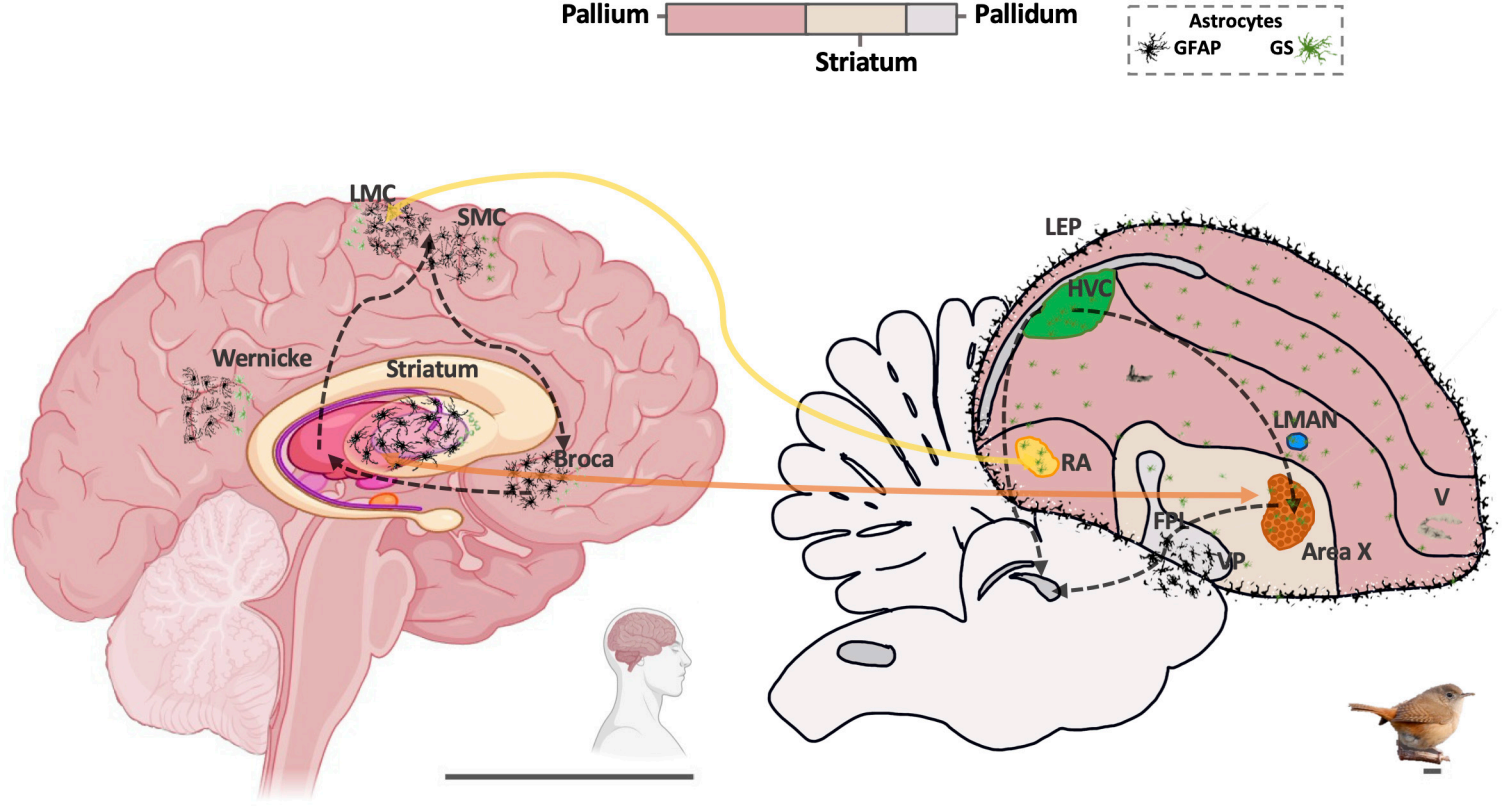
Comparative representation of the main areas associated with vocalization in humans and songbirds. A representative diagram illustrates the homology between the human striatum and Area X in songbirds, as well as between the human Laryngeal Motor Cortex (LMC) and the Robust nucleus of arcopallium (RA) in birds. The figure also shows Glial fibrillary acidic protein (GFAP)-positive astrocytes (black) in human speech-related regions, and in the laminar border of the pallium (LEP) and pallidal territories in songbirds. Glutamine synthetase (GS)-positive astrocytes (green) are present in human speech regions and in nuclei responsible for vocalization in songbirds. Notably, GS-positive astrocytes are broadly distributed across the pallium, whereas GFAP-positive astrocytes appear restricted to specific territories [LEP, vascular domains, and Ventral Pallidum VP/ Fasciculus Prosencephali Lateralis (FPL)].

This study presents, for the first time, a comparative analysis of astrocyte morphology and distribution between humans and the Southern house wren, focusing on brain regions involved in speech and song, respectively. Our results reveal that GFAP-expressing astrocytes in birds exhibit convergent morphology with humans but display a distinct distribution pattern in vocal brain areas. In the Southern house wren telencephalon, GFAP-positive astrocytes formed a laminar pattern restricted to the LEP, perivascular domains, and FPL white matter, paralleling the upper-layer (I–III) astrocyte distribution in the human cortex. Astrocyte density was highest in the human striatum and in the ventral pallidum / FPL of wrens, both basal ganglia territories. GS labeling showed that GFAP marks only a restricted astrocyte subset, whereas GS-positive astrocytes were abundant and homogeneous across the pallium, pallidum, and all vocal nuclei, indicating a broader astroglial scaffold than GFAP alone suggests (Figure 8). Transcriptomic evidence in zebra and Bengalese finches reveals *GLUL* (GS)-high, *GFAP*-low astrocytes in HVC, RA, and Area X, in agreement with our histological findings and indicating that GS marks the main astrocyte population in these songbird regions.

We further compared astrocyte morphology in layer V of the human cortex with analogous regions in the Southern house wren (LEP, vascular pallium, and FPL). This revealed marked interspecies differences in process number, length, soma size, and spatial coverage, with human astrocytes being notably more complex. Despite sharing habitats with humans, the Southern house wren maintains the ability to learn complex songs from a tutor, even in environments enriched with diverse auditory and visual stimuli. GS-based analyses showed that branching complexity was similar between humans and Southern house wrens, indicating that differences mainly reflect the GFAP-positive subset rather than the broader GS population. GS-positive astrocytes showed a uniform organization across regions, while GFAP-positive astrocytes were more region-specific. Human astrocytes expressed all canonical markers, including GFAP, whereas songbird astrocytes showed strong *GLUL* (GS) with only sparse *GFAP*, indicating that GFAP-positive astrocytes represent a specialized cell enriched in humans.

Songbird models have long been used to study the parallels between birdsong learning and human speech acquisition (Beckers et al., 2012; Brainard and Doupe, 2002; Moore and DeVoogd, 2017; Moorman et al., 2015). The zebra finch (*Taeniopygia guttata*), in particular, has provided key insights into the learning and crystallization phases of song (Bailey et al., 2009; Chakraborty et al., 2017; Daou and Margoliash, 2021; Gobes et al., 2010). Comparative transcriptomic studies between humans and zebra finches have identified gene expression similarities between songbird regions (RA/Area X) and human speech areas (LMC/caudate putamen), suggesting possible functional homologies between LMAN/Broca’s and HVC/Wernicke’s areas, even in the absence of shared gene expression (Gedman et al., 2022; Pfenning et al., 2014). In this study, we chose the Southern house wren as our model due to its natural song-learning behavior, ecological relevance, and coexistence with humans. Adult males displayed a broad vocal repertoire, supporting its utility for comparative neurobiology studies of complex communication systems (Camacho-Schlenker et al., 2011; Keenan et al., 2020).

Astrocytes are highly heterogeneous cells in the central nervous system, with morphology and protein expression patterns varying according to brain region and function (Falcone, 2022; Robertson, 2014; Vasile et al., 2017). Markers such as GS, S100, and aldolase have helped distinguish astrocyte subtypes (Anlauf and Derouiche, 2013; Hol and Pekny, 2015; Melzer et al., 2021; Michetti et al., 2019; O’Leary et al., 2020; Pekny and Pekna, 2004). Single-cell transcriptomic studies have found astrocyte-related gene expression in the RA and HVC nuclei of zebra finches and Bengalese finches, suggesting that astrocytes may support synaptic plasticity and song learning. Our findings demonstrate that in humans, GFAP-positive astrocytes are distributed mainly in cortical layers I–III and in the white matter, while in Southern house wrens, GFAP-positive astrocytes are scarce in the major vocal nuclei but present in cortical layer I and in perivascular areas of the pallium. GFAP-positive astrocytes were sparse and mostly confined to vascular and pallial border regions, with only occasional processes entering vocal nuclei (Caterina et al., 2024; Falcone, 2022; PĘkowska et al., 2025). Accordingly, GS astrocytes likely provide the conserved astroglial scaffold for vocal-related circuits in both birds and humans, onto which a more restricted GFAP-positive subtype is superimposed.

Although the song-control nuclei in birds lack the laminar organization of the human cortex, both species exhibit astrocytes in areas possibly involved in barrier or support functions. Given the limited research on avian astrocytes-most of which has used only GFAP and vimentin-we suggest employing additional markers to reveal potential astrocyte subtypes in birds and further clarify their functions.

In humans, we observed a high density of GFAP-positive astrocytes in the striatum—a basal ganglia region rich in white matter. Basal ganglia are known to play a crucial role in speech through their involvement in thalamocortical feedback loops and dopaminergic regulation of motor areas such as the LMC (Fuertinger et al., 2018; Turk et al., 2021). Similarly, in birds, dopamine levels increase in Area X during singing, and lesions in this region cause speech-like deficits in zebra finches (Chen et al., 2014; Kubikova et al., 2014; Sasaki et al., 2006). Based on our data and these studies, we propose that basal ganglia are critical hubs for the learning and production of both speech and song.

Although we did not find GFAP-positive astrocytes in classic vocal nuclei in birds, they were abundantly expressed in the ventral pallidum and FPL-regions implicated in vocal learning. Lesions in the FPL disrupt proper tutor song learning in juveniles, resulting in abnormal adult song patterns (Chen et al., 2019). *In situ* hybridization has shown that this region is rich in GABAergic neurons, suggesting a role in regulating excitatory/inhibitory balance during song learning (Colquitt et al., 2021). Given the white matter richness of these regions, we hypothesize that astrocytes here primarily support metabolic and synaptic functions, including neurotransmitter recycling and neuronal nourishment (Butt et al., 2014; Köhler et al., 2023; Lundgaard et al., 2014).

In humans, astrocytes display classical morphologies, protoplasmic in gray matter and fibrous in white matter, reflecting well-established regional specialization (Köhler et al., 2021; Kol and Goshen, 2021). Comparative work shows that rodent astrocytes tend to be simpler (Zhang et al., 2016), and that environmental factors such as enrichment can modulate branching and structural complexity (Viola et al., 2009). Across vertebrates, astrocyte diversity reflects deep evolutionary roots: GS-expressing astroglia represent an ancient, conserved lineage supporting core metabolic functions, whereas GFAP-rich phenotypes expanded later in amniotes and primates as more specialized, regionally tuned subtypes (Falcone, 2022; PĘkowska et al., 2025). Astrocyte morphology in birds remains incompletely described, though seasonal changes in astrocyte density have been reported in canaries (Kafitz et al., 1999; Nottebohm et al., 1986). In our study, Southern house wren astrocytes showed clear regional differences, with FPL astrocytes exhibiting more elaborate processes than those in LEP or perivascular territories. Nonetheless, their overall size, morphology, and process coverage remained less complex than human layer V astrocytes. These observations align with broader evolutionary patterns in which conserved GS-positive astrocytes provide a foundational glial scaffold, while GFAP-positive subtypes exhibit species- and region-specific differentiation.

This study has several limitations. We did not distinguish protoplasmic from fibrous astrocytes, and the sample size was modest (*n* = 5 per species), though all wrens were reproductively active adults collected under comparable eco-physiological conditions. Seasonal effects on GFAP expression cannot be fully ruled out. Because GFAP labels only a restricted avian subset, low signal in vocal nuclei should be interpreted as sparse labeling, not absence. Orthology analysis and immunofluorescence show that GS and GFAP antibodies recognize astrocytes in both humans and Southern house wren, with GS–GFAP colocalization in perivascular regions; GLUL/GS expression in two songbird scRNA-seq datasets supports this. However, wren-specific GFAP epitope validation remains incomplete. The integrated scRNA-seq datasets come from non-tropical songbirds and from human frontal cortex and striatum, which may not fully represent vocal-motor regions but still provide informative comparative context. Finally, examining only one vocal-learning bird and one mammalian species—without non–vocal-learning controls—precludes separating species-specific traits from vocal-learning features; thus, our interpretations are strictly anatomical correlations, and broader comparative sampling will be required to test phylogenetic or behavioral explanations.

## Conclusion

In conclusion, GS-positive astrocytes represent the conserved astroglial framework shared by birds and humans, whereas GFAP-positive astrocytes, though present and detectable in wrens, are more abundant and structurally complex in human speech circuits, reflecting both shared substrates for vocal learning and species-specific astrocyte specialization.

## Data Availability

The original contributions presented in the study are included in the article/Supplementary material, further inquiries can be directed to the corresponding author. Cross-species integrative bioinformatics analyses can be found here: https://github.com/HernanHoyosUdeA/Avian-Human-sc-RNA-seq.

## Supplementary material

Supplementary Figure 1 Localization of vocal brain areas in the Southern house wren across mediolateral bregma levels. **(A)** Schematic of mediolateral slices used in 3D reconstruction. **(B)** Color-coded locations of major vocal nuclei: Lateral magnocellular nucleus of the anterior nidopallium (LMAN) (blue), Area X (orange), HVC (green), and Robust nucleus of arcopallium (RA) (yellow). Nissl-stained tissue is shown for bregma levels 420, 690, 1,140, 1,350, 1,890, 2,010, and 2,880 μm from the midline. Scale bar: 5 mm.

Supplementary Figure 2 GFAP and S100β immunolabeling and regional GS distribution in the Southern house wren and humans. (**A**, top) IF images of Glial fibrillary acidic protein (GFAP)-positive astrocytes in Laminar edge of the pallium (LEP), vascular pallium, and Fasciculus Prosencephali Lateralis (FPL) regions. (**A**, middle) Corresponding immunohistochemistry (IHC) images of the same regions. (**A**, bottom) Skeletonized reconstructions of individual astrocytes. Scale bars: 25 μm (IF 60X); 10 μm (IHC 100x-objective images). **(B)** Maximum processes distance from soma is greatest in Ventral Pallidum (VP) / Fasciculus Prosencephali Lateralis (FPL) astrocytes; significant differences are detected between LEP–FPL (“*p* = 0.0156”) and vascular–FPL (“*p* = 0.0021”). Statistical analyses were performed using one-way ANOVA with Tukey’s multiple comparisons test. **(C)** IF negative controls images for double staining for GS (green) and GFAP (red) for humans and different songbird areas. **(D)** Immunofluorescence (IF) images showing nuclei (blue), GFAP (green), S100β (red), and the merged composite (40X). These images highlight sparse GFAP-positive and S100β-positive astrocytic elements near the laminar edge of the pallium (LEP). Scale bar: 50 μm. (**E**, top) Quantification of Glutamine synthetasxe (GS) levels, expressed as percentage of labeled area, across human speech-related cortical and striatal regions, each represented by distinct colors. (**E**, down) Quantification of GS-positive area along the Southern house wren pallium, extending from LEP toward basal ganglia territories (FPL), illustrating the distribution of GS expression across the telencephalic axis. Significance: **p* < 0.05, ***p* < 0.01, ****p* < 0.001.

Supplementary Figure 3 Cell-type annotation and astrocyte marker expression. **(A)** Dot plot of marker genes used to annotate avian cell classes from HVC, Robust nucleus of arcopallium (RA), and Area X; circle size indicates the fraction of cells in each group, and color denotes average expression. Markers include *SOX6/SLC1A2/MAP2/CUX2* (excitatory), *GAD1/GAD2* (inhibitory), *SLC1A2/SLC1A3* (astrocytes), *FTH1* (microglia), *MBP/PLP1* (oligodendrocytes), *SOX6/PDGFRA* (OPCs), *FLT1* (vascular), and *SOX4* (immature). **(B)** UMAP showing regional contribution from the avian nuclei: HVC (20,870 cells), Area X (14,153), RA (5,032); bottom panel shows species contribution: zebra finch (38,791) and Bengalese finch (1,264). **(C)** UMAP of avian astrocytes showing *GLUL, GFAP, AQP4*, and *S100B* expression patterns. **(D)** Dot plot of human cell-type markers, with circle size representing fraction of cells and color indicating mean expression. Markers include *FOXP2/DRD1/GAD2* (MSN1), *DRD2/GAT2* (MSN2), *DRD2/FOXP2/GAT2* (MSN-caudate), *GAT1/GAT2* (inhibitory), *SLC17A7/CUX2* (excitatory), *AQP4/GFAP* (astrocytes), *P2RY12* (microglia), *MBP* (oligodendrocytes), *VCAN* (OPCs), and *CLDN5* (vascular). **(E)** UMAP showing human regional contributions: prefrontal cortex (38,787 nuclei), putamen (27,598), and caudate (36,902). **(F)** UMAP of the human astrocyte subcluster showing *GLUL, GFAP, AQP4*, and *S100B* expression across PFC–TNC^+^, PFC–TNC^+^, STR–TNC^+^, and STR–TNC^+^ astrocytes.

Supplementary Figure 4 Expression levels and proportion of astrocyte marker-expressing cells. **(A)** Boxplots showing the percentage of finches astrocytes with expression values greater than zero for *GFAP, GLUL*, and *S100B*. **(B)** Violin plots showing the distribution of log-normalized expression for the same markers across finches astrocytes. Internal boxplots indicate the median and interquartile range, and individual points represent single-cell expression values. Finch datasets correspond to *n* = 2 Bengalese finches and *n* = 3 zebra finches. **(C)** Boxplots showing the percentage of human astrocytes with expression values greater than zero for *GFAP, GLUL*, and *S100B*. **(D)** Violin plots showing the distribution of log-normalized expression for the same genes in astrocytes from human control samples. Boxplots within violins indicate the median and interquartile range. Prefrontal cortex, putamen, and caudate datasets (*n* = 6, *n* = 6, and *n* = 8, respectively).
