# Supplementary figures and images for: Differential morphology and distribution of GFAP astrocytes in vocal brain circuit in a songbird Southern house wren and humans
Source: Front Neuroanat. 2026 Feb 6;20:1606172. doi: 10.3389/fnana.2026.1606172 (PMC12920451; doi:10.3389/fnana.2026.1606172)

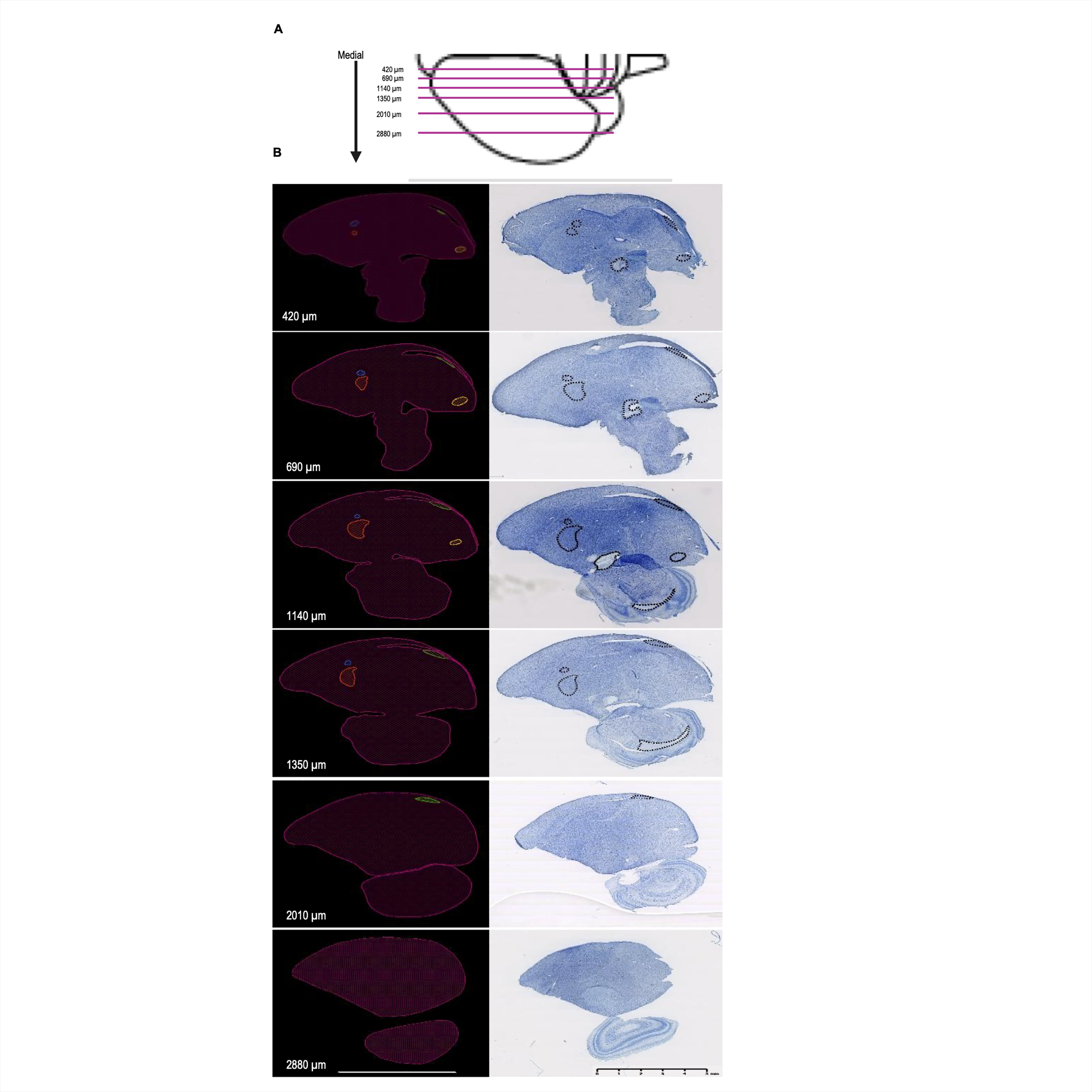

Supplement: Supplementary Figure 1 — Localization of vocal brain areas in the Southern house wren across mediolateral bregma levels. (A) Schematic of mediolateral slices used in 3D reconstruction. (B) Color-coded locations of major vocal nuclei: Lateral magnocellular nucleus of the anterior nidopallium (LMAN) (blue), Area X (orange), HVC (green), and Robust nucleus of arcopallium (RA) (yellow). Nissl-stained tissue is shown for bregma levels 420, 690, 1,140, 1,350, 1,890, 2,010, and 2,880 μm from the midline. Scale bar: 5 mm. [file Image_1.tiff]

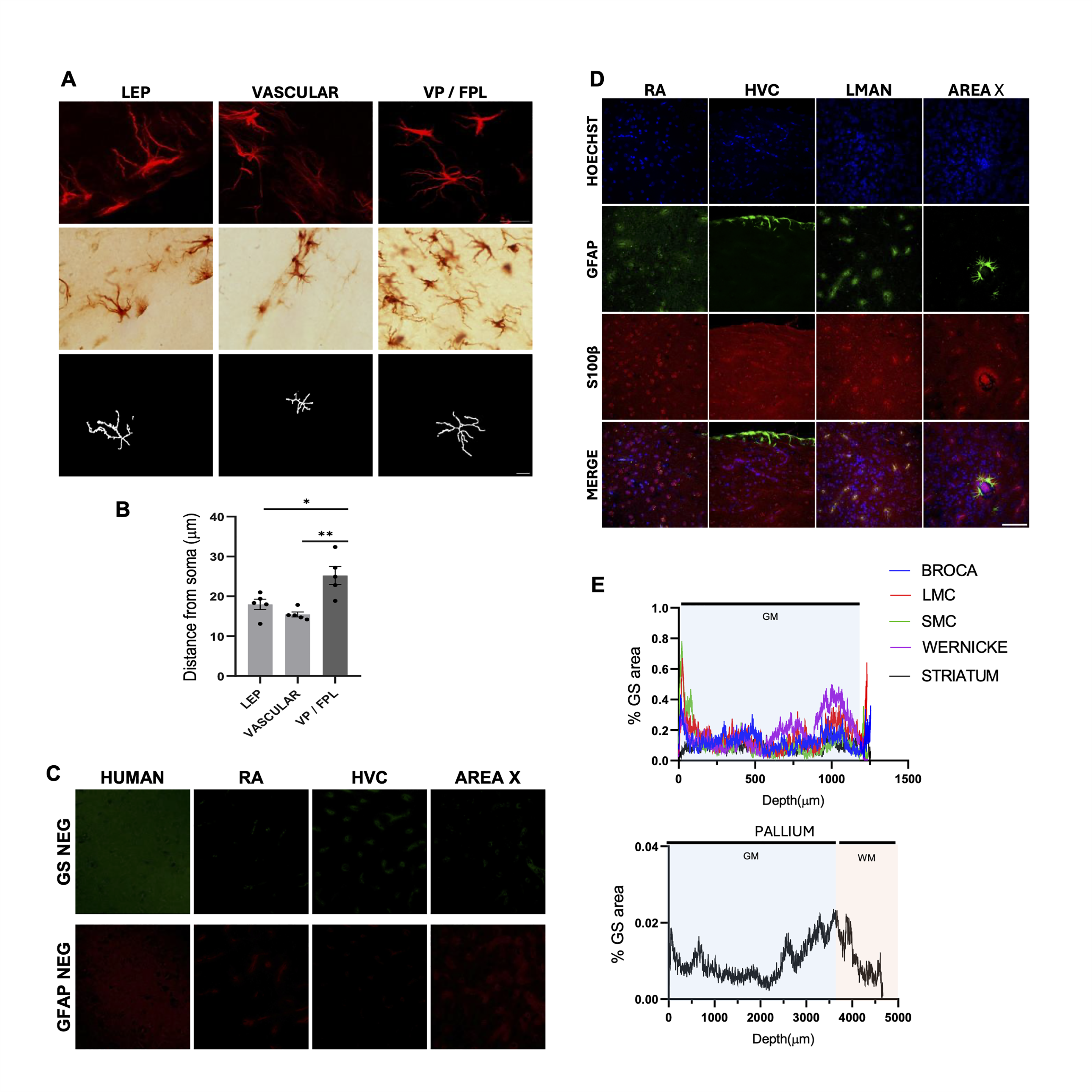

Supplement: Supplementary Figure 2 — GFAP and S100β immunolabeling and regional GS distribution in the Southern house wren and humans. (A, top) IF images of Glial fibrillary acidic protein (GFAP)-positive astrocytes in Laminar edge of the pallium (LEP), vascular pallium, and Fasciculus Prosencephali Lateralis (FPL) regions. (A, middle) Corresponding immunohistochemistry (IHC) images of the same regions. (A, bottom) Skeletonized reconstructions of individual astrocytes. Scale bars: 25 μm (IF 60X); 10 μm (IHC 100x-objective images). (B) Maximum processes distance from soma is greatest in Ventral Pallidum (VP) / Fasciculus Prosencephali Lateralis (FPL) astrocytes; significant differences are detected between LEP–FPL (“p = 0.0156”) and vascular–FPL (“p = 0.0021”). Statistical analyses were performed using one-way ANOVA with Tukey’s multiple comparisons test. (C) IF negative controls images for double staining for GS (green) and GFAP (red) for humans and different songbird areas. (D) Immunofluorescence (IF) images showing nuclei (blue), GFAP (green), S100β (red), and the merged composite (40X). These images highlight sparse GFAP-positive and S100β-positive astrocytic elements near the laminar edge of the pallium (LEP). Scale bar: 50 μm. (E, top) Quantification of Glutamine synthetasxe (GS) levels, expressed as percentage of labeled area, across human speech-related cortical and striatal regions, each represented by distinct colors. (E, down) Quantification of GS-positive area along the Southern house wren pallium, extending from LEP toward basal ganglia territories (FPL), illustrating the distribution of GS expression across the telencephalic axis. Significance: *p < 0.05, **p < 0.01, ***p < 0.001. [file Image_2.tiff]

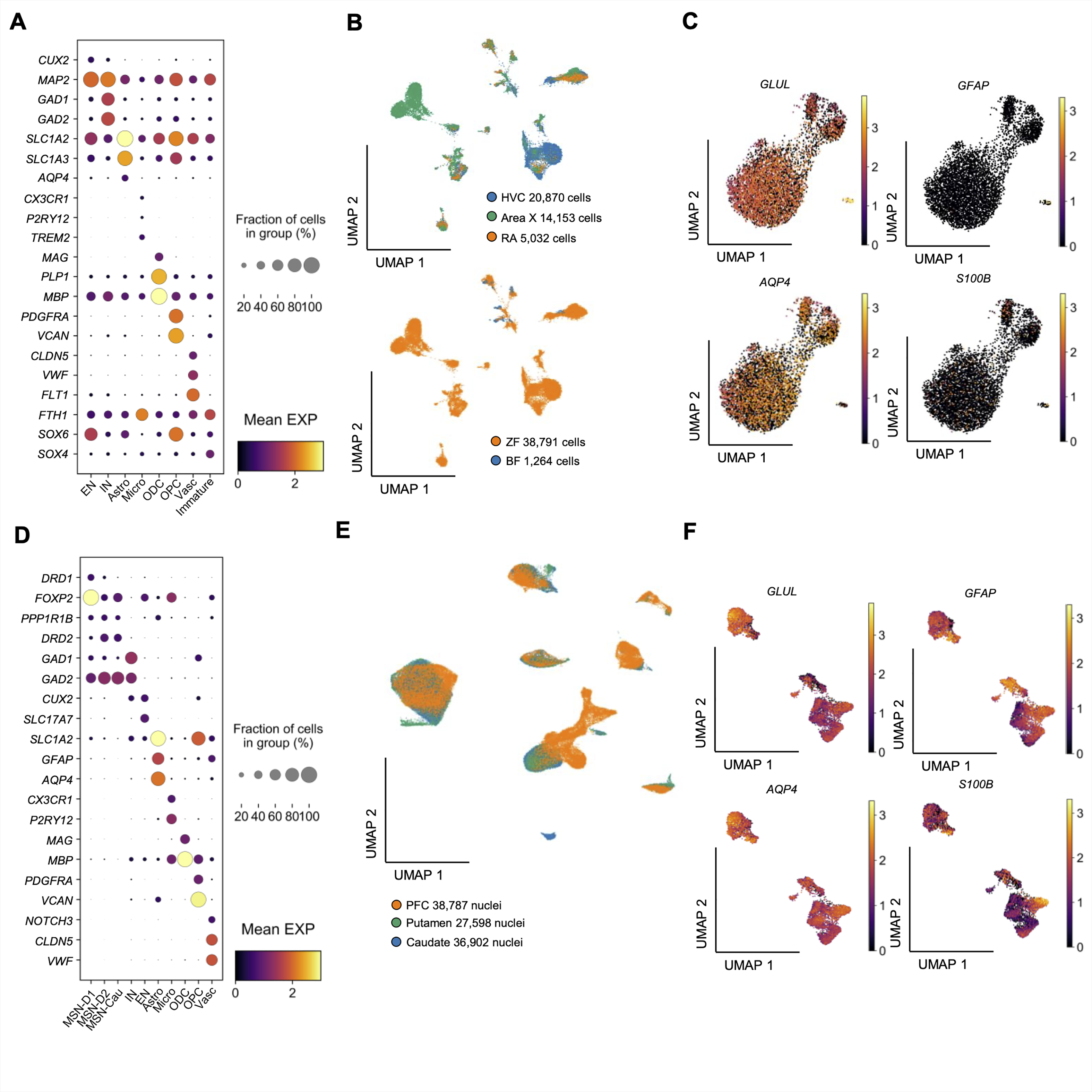

Supplement: Supplementary Figure 3 — Cell-type annotation and astrocyte marker expression. (A) Dot plot of marker genes used to annotate avian cell classes from HVC, Robust nucleus of arcopallium (RA), and Area X; circle size indicates the fraction of cells in each group, and color denotes average expression. Markers include SOX6/SLC1A2/MAP2/CUX2 (excitatory), GAD1/GAD2 (inhibitory), SLC1A2/SLC1A3 (astrocytes), FTH1 (microglia), MBP/PLP1 (oligodendrocytes), SOX6/PDGFRA (OPCs), FLT1 (vascular), and SOX4 (immature). (B) UMAP showing regional contribution from the avian nuclei: HVC (20,870 cells), Area X (14,153), RA (5,032); bottom panel shows species contribution: zebra finch (38,791) and Bengalese finch (1,264). (C) UMAP of avian astrocytes showing GLUL, GFAP, AQP4, and S100B expression patterns. (D) Dot plot of human cell-type markers, with circle size representing fraction of cells and color indicating mean expression. Markers include FOXP2/DRD1/GAD2 (MSN1), DRD2/GAT2 (MSN2), DRD2/FOXP2/GAT2 (MSN-caudate), GAT1/GAT2 (inhibitory), SLC17A7/CUX2 (excitatory), AQP4/GFAP (astrocytes), P2RY12 (microglia), MBP (oligodendrocytes), VCAN (OPCs), and CLDN5 (vascular). (E) UMAP showing human regional contributions: prefrontal cortex (38,787 nuclei), putamen (27,598), and caudate (36,902). (F) UMAP of the human astrocyte subcluster showing GLUL, GFAP, AQP4, and S100B expression across PFC–TNC+, PFC–TNC+, STR–TNC+, and STR–TNC+ astrocytes. [file Image_3.tiff]

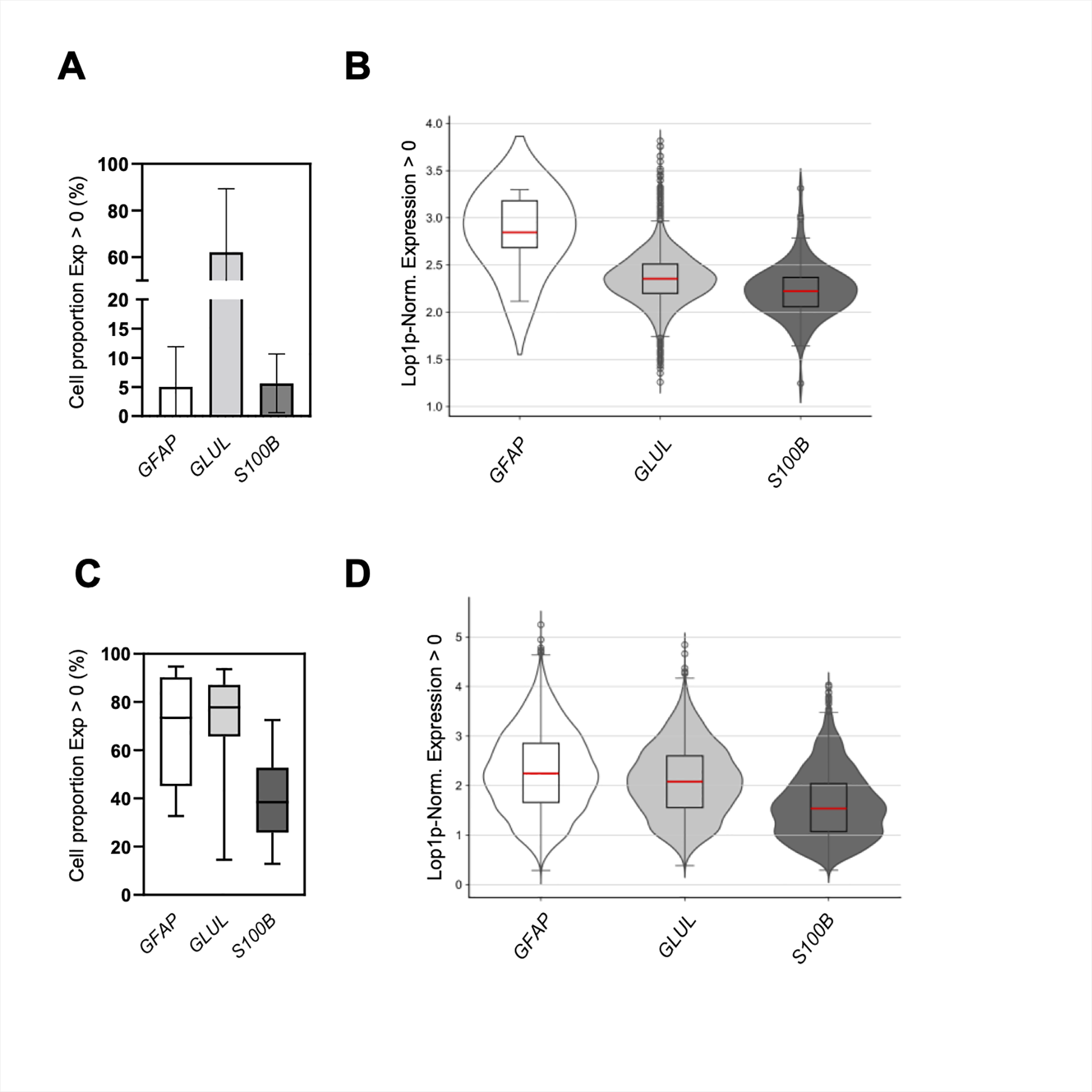

Supplement: Supplementary Figure 4 — Expression levels and proportion of astrocyte marker-expressing cells. (A) Boxplots showing the percentage of finches astrocytes with expression values greater than zero for GFAP, GLUL, and S100B. (B) Violin plots showing the distribution of log-normalized expression for the same markers across finches astrocytes. Internal boxplots indicate the median and interquartile range, and individual points represent single-cell expression values. Finch datasets correspond to n = 2 Bengalese finches and n = 3 zebra finches. (C) Boxplots showing the percentage of human astrocytes with expression values greater than zero for GFAP, GLUL, and S100B. (D) Violin plots showing the distribution of log-normalized expression for the same genes in astrocytes from human control samples. Boxplots within violins indicate the median and interquartile range. Prefrontal cortex, putamen, and caudate datasets (n = 6, n = 6, and n = 8, respectively). [file Image_4.tiff]
